# Skeleton-Controlled pDNA Delivery of Renewable Steroid-Based Cationic Lipids, the Endocytosis Pathway Analysis and Intracellular Localization

**DOI:** 10.3390/ijms19020369

**Published:** 2018-01-26

**Authors:** Ruilong Sheng, Zhao Wang, Ting Luo, Amin Cao, Jingjing Sun, Joseph M. Kinsella

**Affiliations:** 1 Key Laboratory of Synthetic and Self-Assembly Chemistry for Organic Functional Molecules, Shanghai Institute of Organic Chemistry, Chinese Academy of Sciences, Lingling Road 345, Shanghai 200032, China; wangzhaosioc@163.com (Z.W.); luoting@sioc.ac.cn (T.L.); feliciasun23@gmail.com (J.S.); 2 Department of Bioengineering, McGill University, 817 Sherbrook Street, Montréal, QC H3A0C3, Canada; 3 CQM—Centro de Química da Madeira, Universidade da Madeira, Campus da Penteada, 9000-390 Funchal, Portugal

**Keywords:** steroid, gene delivery, serum-compatible, structure–function relationships, endocytosis pathway

## Abstract

Using renewable and biocompatible natural-based resources to construct functional biomaterials has attracted great attention in recent years. In this work, we successfully prepared a series of steroid-based cationic lipids by integrating various steroid skeletons/hydrophobes with (*l*-)-arginine headgroups via facile and efficient synthetic approach. The plasmid DNA (pDNA) binding affinity of the steroid-based cationic lipids, average particle sizes, surface potentials, morphologies and stability of the steroid-based cationic lipids/pDNA lipoplexes were disclosed to depend largely on the steroid skeletons. Cellular evaluation results revealed that cytotoxicity and gene transfection efficiency of the steroid-based cationic lipids in H1299 and HeLa cells strongly relied on the steroid hydrophobes. Interestingly, the steroid lipids/pDNA lipoplexes inclined to enter H1299 cells mainly through caveolae and lipid-raft mediated endocytosis pathways, and an intracellular trafficking route of “lipid-raft-mediated endocytosis→lysosome→cell nucleic localization” was accordingly proposed. The study provided possible approach for developing high-performance steroid-based lipid gene carriers, in which the cytotoxicity, gene transfection capability, endocytosis pathways, and intracellular trafficking/localization manners could be tuned/controlled by introducing proper steroid skeletons/hydrophobes. Noteworthy, among the lipids, Cho-Arg showed remarkably high gene transfection efficacy, even under high serum concentration (50% fetal bovine serum), making it an efficient gene transfection agent for practical application.

## 1. Introduction

In recent decades, transformation of renewable and biocompatible natural resources [1] into various functional products including chemical reagents, pharmaceutics, oils and fuels, and new functional materials has been highly focused for nurturing the sustainable development. Steroids, a large natural lipids family known as “keys of life”, play vital roles including membrane formation, hormone metabolism and cell signal transduction in organelles. Some steroidal compounds possess special physicochemical features such as hydrophobicity, rigidity, mesogenic behaviors, etc., which make them functional building blocks for the construction of supramolecular architectures [2], soft matters and biomaterials [3].

As a hot spot in biomaterial research, developing new cationic lipids as non-viral gene (DNA, oligo DNA, siRNA, etc.) carriers toward gene therapy has achieved increasing attention in the past few decades [4,5]. A satisfactory lipid gene carrier should be highly biocompatible [6] and efficiently load and release therapeutic gene substances [7] into target cells. Recent researches revealed that the introduction of some steroid hydrophobes to gene/drug carriers could enhance drug loading capacity and delivery efficiency [8], improve estrogen receptor (ER) affinity [9], lower cytotoxicity and membrane disruption [10], etc. In the last decade, cholesterol was the most commonly-used steroidal compound for the construction of functional gene/drug [11] carriers. For example, Bhattacharya et al. developed a series of cholesterol cationic lipids [12] and Gemini-lipids [13,14,15,16,17] with remarkably high gene transfection efficiency and transfected p53-EGFP-C3 plasmid DNA to induce tumor apoptosis [18]. Rana et al. [19] prepared some cholesterol-hybridized cationic lipids with enhanced siRNA delivery efficiencies and lower cytotoxicity. Zenkova et al. [20,21,22] disclosed series of cholesterol cationic lipids modified with heterocyclic (pyridine, methyl imidazole, etc.) or polyamine headgroups that have low cytotoxicity and high transfection efficiency, and some cholesterol-based cationic glucosidal lipids have similar properties [23]. In our earlier work, we prepared a series of bioreduction-responsive cholesterol disulfide cationic (CHOSS) lipids [24], which possessed low cytotoxicity, high pDNA transfection efficiency, and perinucleic localization effect. Afterward, we studied the contribution of headgroups/linkages of some cholesterol-based cationic lipids on their gene delivery manners, finding that the physicochemical features and gene transfection properties greatly relied on the cationic amino-acid headgroups rather than chemical linkages [25]. Except cholesterol, some other steroidal compounds such as diosgenin (a phytosteroid sapogenin used in the preparation of different steroids, e.g., cortisone), bile acids, etc. were employed to construct lipid gene carriers. For example, Regen et al. developed series of “molecular umbrella” amphiphiles [26] and disulfide-containing bile acid-siRNA conjugates [27] for intracellular siRNA delivery. Yu et al. [28,29,30] synthesized some diosgenin-based cyclen cationic lipids with the merit of low cytotoxicity and high transfection efficiency. In a previous work, we synthesized some cholesterol/lithocholate-based cationic lipids via CuAAC “Click” approach and disclosed that their gene transfection efficiency relied greatly on the steroid structures [31]. However, the working performance, such as biocompatibility, transfection efficiency, serum-compatibility, and membrane permeability, of most steroid-based gene carriers is still far from their maximum value, especially far below their natural virus counterparts. Moreover, the endocytosis manner and intracellular trafficking/localization of most steroid-based gene delivery systems is not very clear yet. 

It has been known that the endocytosis mechanism greatly affects the intracellular gene transfection efficacy and subcellular distribution of gene carriers [32]. For the endocytosis pathways of steroid-containing gene carriers, only a few cases were investigated. Bae et al. [33] found that clathrin-mediated endocytosis is the dominant pathway for cholesterol-based (CHOL-E) liposomes. While Pozzi et al. [34] disclosed that macropinocytosis is the only endocytosis pathway of a cholesterol cationic lipid (DC-Chol) containing multi-component envelope-type nanoparticle system (MENS). Besides, Jeong et al. [35] disclosed clathrin, caveolae and pinocytosis pathways involved in the cellular uptake mechanism of hydrophobic 5β-cholanic acid containing glycolchitosan (HGC) nanoparticles. However, to date, the correlation between chemical structures and endocytosis pathways for most of the steroid-based gene delivery systems remains unclear.

Based on our previous works [24,25,31], we continue exploring the lipid structure–gene delivery function relationships and further understanding the roles that steroidal hydrophobes played in gene delivery manners, such as cellular uptake, transfection efficiency, serum-compatibility, endocytosis pathway, and intracellular localization. In this work, we synthesized a series of new steroid-based cationic lipids with the common arginine headgroups and 6-amino-caprolate linkers but different steroid hydrophobes/skeletons. The pDNA binding affinities of the newly synthesized steroid-based lipids were examined by agarose gel retardation assays. The average particle sizes, surface potentials and morphologies of the steroid-based lipids/pDNA lipoplexes were investigated by dynamic laser light scattering (DLS) and transmission electron microscopy (TEM), respectively. Cytotoxicities of the lipids were evaluated by a thiazoyl blue tetrazolium bromide (MTT) method, and their gene transfection efficiency were investigated by Luciferase and EGFP expression assays in the presence/absence of serum with multiple cell lines. The endocytosis pathway was studied by luciferase expression assays with specific endocytosis inhibitors. The cellular uptake efficiency was evaluated by flow cytometry. Finally, the intracellular localization of the steroid-based lipids/Cy3-pDNA lipoplexes was observed under fluorescence microscopy.

## 2. Experimental Section

### 2.1. Materials

Cholesterol (97.0%) was purchased from Sinopharm Chemical Reagent Co., Ltd. (Shanghai, China) 2H-Cholesterol (Cholestanol, 95.0%), Diosgenin (95.0%) and Tigogenin (98.0%) were purchased from Sigma-Aldrich (St. Louis, MO, USA) and utilized as received. Boc-protected 6-amino-caproic acid was prepared according to literature. BOC-Arg-OH·HCl·H_2_O (99.0%) was purchased from GL Biochemical Co., Ltd. (Shanghai, China). N-(3-dimethyl-aminopropyl)-N′-ethylcarbodiimide hydrochloride (EDC·HCl, 99.0%), 4-dimethyl amino pyridine (DMAP, 99.0%) and trifluoroacetic acid (TFA, 99.0%) were provided by Shanghai Sinopharm Chemical Reagent Co., Ltd. Other reagents (Shanghai, China) and solvents were analytical grade and utilized as received.

Agarose was purchased from Gene Tech. (Shanghai, China). Ethidium bromide (EB) (95.0%) and branched poly (ethylene imine) (bPEI-25k, *M*_w_ = 25,000) were supplied by Sigma-Aldrich. Lipofectamine2000(Lipo2000) was received from Invitrogen (Carlsbad, CA, USA) (Lot No. 637822). Thiazoyl blue tetrazolium bromide (MTT) was bought from Biobasic Inc., (Markham, ON, Canada). Luciferase assay and bicinchoninic acid (BCA) protein quantization kits were, respectively, supplied by Promega (Madison, WI, USA) and Applygen Technologies (Beijing, China). Phosphate buffer solution (1 × PBS) (0.1 M), DMEM medium and 10% fetal bovine serum (FBS) were purchased from Hangzhou Genom Co., Ltd. (Hangzhou, China). The 96-well and 24-well microplates, and 50 mL cultivation flasks were purchased from Corning Co., Ltd. (Corning, NY, USA) Human lung cancer (H1299) and Human epithelial cervical cancer (HeLa) cell lines were obtained from Yuhong Xu’s lab at Shanghai Jiaotong University. The endocytosis specific inhibitors, chlorpromazine hydrochloride (99.2%), genistein (99.0%), Nocodazole (99.0%) and methyl-β-cyclodextrin (99.0%), were purchased from Sigma-Aldrich. Amiloride (98.0%) was purchased from Fluorochem Ltd. (Old Glossop, UK). Label IT^®^ Tracker™ intracellular nucleic acid (Cy3-labeled pDNA) localization kits including Trans IT^®^-LT1 transfection reagent were purchased from Mirus Bio LLC (Madison, WI, USA). Lysotracker green DND-26 was received from Invitrogen (Cat#L7526). DAPI was received from Roche (Cat#70217321, Basel, Switzerland). In addition, all the other reagents and chemicals were of analytical grade and were utilized as received.

### 2.2. Synthesis Procedures of the Steroids-Based Cationic Lipids

Synthesis procedures of the steroid-based cationic lipids and their important intermediates are described in detail in Supplementary Materials S1.

### 2.3. Nuclear Magnetic Resonance (NMR) and Mass Spectral Measurements 

^1^H nuclear magnetic resonance (NMR) spectra were characterized on a Varian VXR-300 Fourier transform NMR spectrometer at 300.0 MHz for proton nuclei at ambient temperature; ^13^C NMR spectra were determined on a Bruker Avance NMR spectrometer at 100.0 MHz for the ^13^C nuclei; and tetramethylsilane (TMS) was utilized as the internal chemical shift reference. Mass spectra (ESI-MS) were routinely measured on a Varian SATURN 2000 instrument (Palo Alto, CA, USA).

### 2.4. Agarose Gel Retardation Assay

pDNA binding affinity of the new steroid-based cationic lipids was determined by agarose gel retardation assay [31]. The solution of steroid-based cationic lipids/pDNA lipoplexes were firstly prepared under a predetermined +/− charge ratio with 0.01 M PBS and 1.0 µg of pDNA in 20 µL of pure water and kept incubation for 30 min. Then, the lipoplex solution was loaded onto 1% agarose gel (*w*/*v*) with EB (0.5 µg/mL in gel) and TAE buffer (pH = 7.4), and the naked pDNA was used as the control. After the gel electrophoresis under +100 V for 1 h, the retardant of pDNA was recorded on a UV transilluminator (UVP, Upland, CA, USA) benchtop 2UV transilluminator system. 

Stability of the steroid-based cationic lipids/pDNA lipoplexes were analyzed by agarose gel retardation assay. The lipoplex solution of each steroid lipid was prepared according to the aforementioned method under the N/P charge ratios of 15 and incubated for 40 min. After that, various amount of heparin solution (1 mg/mL) was added into each lipoplex solution and further incubated for 40 min. Then, each solution was loaded onto 1% agarose gel (*w*/*v*) containing EB dye (0.5 µg/mL) in TAE buffer solution (pH = 7.4) and run for 40 min at 100 V. The plasmid DNA retardation was illuminated and photographed on a UVP.

### 2.5. Particle Sizes and ζ Potentials of the Steroid-Based Cationic Lipids/pDNA Lipoplexes

Average particle sizes and ζ potentials of the Steroid-based cationic lipids/pDNA lipoplexes were analyzed on a Malvern Zetasizer Nano ZS90 (Worchestershire, UK) at room temperature. The lipoplex solutions were first prepared by mixing steroid-based cationic lipids and pDNA (2.0 µg/mL) under diverse +/− charge ratios in 1 mL pure water, and then laser light at λ = 633 nm was employed at a fixed scattering angle of 90° for the nanoparticle size analyses.

To investigate the effect of serum, the as-prepared steroid-based cationic lipids/pDNA lipoplexes were incubated with BSA (1 mg/mL) solution for 10 min, and then the average particle sizes and ζ potentials were analyzed on a Malvern Zetasizer Nano ZS90 at ambient temperature.

### 2.6. Morphologies of the Steroid-Based Cationic Lipids/pDNA Lipoplexes by TEM

Morphology of the steroid-based cationic lipids/pDNA lipoplexes were characterized at room temperature on a transmittance electronic microscopy (TEM, JEOL-1230, JEOL Co., Ltd., Tokyo, Japan) with an acceleration voltage of 80 KV. The lipoplexes solution were firstly prepared by mixing steroid-based cationic lipids and pDNA (5.0 µg/mL) under +/− charge ratios of 15 in 1 mL of pure water, and then the lipoplex samples for TEM analysis were dropped onto a 300-mesh carbon-coated copper grid and air-dried at room temperature.

### 2.7. Cytotoxicity of the Steroid-Based Cationic Lipids by MTT and CCK-8 Assays

Cytotoxicity of the steroid-based cationic lipids were evaluated in H1299 (by MTT assay) [31] and HeLa cell lines (by CCK-8 assay) [36]. Cells were firstly seeded into 96-well microplates at 4 × 10^3^ cells per well, and were cultivated under 37 °C and 5% CO_2_ for 24 h in 100 µL DMEM medium (with 10% FBS). Subsequently, the medium was replaced with fresh DMEM medium (with 10% FBS), and then each steroid-based cationic lipid under varies concentrations was individually added into the wells and further incubated for another 24 h. After that, 20 µL of 5.0 mg/mL MTT (or 10 µL of CCK-8) was added into each well. For MTT assay, after 4 h incubation, the medium was removed, and DMSO (100 µL/well) was added to dissolve the formed MTT formazan with gently shaking for 10 min, then each sample with six replicates (*n* = 6) was analyzed on a microplate reader (BioTek, ELX800, Winooski, VT, USA) at λ = 490 (λ_ref_ = 630 nm). For CCK-8 assay, after 3 h incubation, each sample with six replicates (*n* = 6) was directly analyzed on a microplate reader (BioTek, ELX800) at λ = 490 (λ_ref_ = 630 nm). Commercially available branched-polyethylenimine (bPEI-25k) was applied as a reference in both MTT and CCK-8 cytotoxicity assays.

### 2.8. Luciferase Gene Transfection Assay in the Presence and Absence of Serum

Gene transfection efficiency of the steroid-based cationic lipids were evaluated by luciferase gene transfection assay [37]. H1299 and HeLa cells were firstly seeded into 24-well microplates (5 × 10^5^ cells/well) and incubated under 37 °C and 5% CO_2_ with DMEM (10% FBS) for 24 h. Then, lipoplex solutions were prepared by mixing the steroid-based cationic lipids and luciferase pDNA (1.0 µg/well) in 200 µL FBS-free DMEM medium or DMEM with 10%, 20% and 50% FBS at a predetermined +/− charge ratio and incubated for 30 min. The commercially available bPEI-25k (± = 10) and Lipofectamine2000 (Lipo2000, *w*/*w* = 3) were used as references. Then, the prepared lipoplex/complex solutions were added into the 24-well plates and incubated for 4 h (0% FBS) or 24 h (10%, 20%, and 50% FBS). For the transfection with 0% FBS, the medium was replaced with 200 µL fresh DMEM medium (with 10% FBS) and further incubated for 20 h. Consequently, the Luciferase transfection assays were conducted in accordance to the protocol of Promega Luciferase assay system. Finally, total Luciferase protein was measured with BCA assay kit (Applygen Technologies Inc., Beijing, China), and relative light unit per milligram of Luciferase protein (RLU/mg) was calculated to evaluate the cell transfection efficacy of the steroid-based cationic lipids (*n* = 3).

### 2.9. Intracellular Uptake of the Steroid-Based Cationic Lipids/Cy3-pDNA Lipoplexes Measured by Flow Cytometry

Cy3-labeled pDNA (Cy3-pDNA) was prepared according to the protocol of Label IT^®^ Tracker™ intracellular nucleic acid localization kits (Mirus Bio LLC) [24]. The steroid-based cationic lipids/Cy3-pDNA complexes was prepared by mixing the cationic lipids with Cy3-pDNA (200 ng/mL, ± = 10) and incubated at room temperature for 30 min. Moreover, the bPEI-25k/Cy3-pDNA (± = 10) and Lipo2000/Cy3-pDNA (*w*/*w* = 3) were used as positive references.

HeLa cells were seeded into 6-well microplates at a density of 3 × 10^5^ cells/well and cultivated in DMEM medium (with 10% FBS) for 24 h. Subsequently, the cells were cultured with fresh serum- free medium, treated with the as-prepared cationic lipids/Cy3-pDNA lipoplexes, and incubated for 4 h. Thereafter, the cells were washed with 1 × PBS for three times and digested by trypsinization. After centrifugation, the harvested cells were re-suspended in 1 mL 1 × PBS solution and were transferred to a flow cytometry (BD FACS Calibur, Mountain View, CA, USA, set as 10,000 cells for each sample). During the FACS analysis, the HeLa cells were gated by sideward scatter versus forward scatter (SSC/FSC) plots, and the Cy3 fluorescence intensities were recorded in the FL2-H channel.

### 2.10. Luciferase Transfection Assays under Various Endocytosis Inhibitors [38]

H1299 cells were seeded in 24-well plates (5 × 10^5^ cells/well) and incubated in culture medium (DMEM, with 10% FBS) at 37 °C with 5% CO_2_ for 24 h. The endocytosis-specific inhibitors, including: Chlorpromazine (CPZ, clathrin-mediated endocytosis pathway inhibitor, 3.0 µg/mL), Genistein (GENI, caveolae-mediated endocytosis pathway inhibitor, 50.0 µg/mL), Amiloride (AMIL, micropinocytosis pathway inhibitor, 2.3 µg/mL), Nocodazole (NOCO, microtubule-assisted phagocytosis inhibitor, 2.0 µg/mL) and methyl-β-cyclodextrin (MBCD, lipid-raft pathway inhibitor, 10.0 µg/mL), were separately added into the H1299 cells, and further incubated for 1 h [38]. Thereafter, the medium was replaced with fresh serum-free culture medium, and the as-prepared steroid-based cationic lipids/pDNA lipoplexes (pDNA 1.0 µg/well, ± = 10) were added and incubated at 37 °C for 4 h. Subsequently, the medium was replaced with fresh medium containing 10% FBS and further incubated for 24 h, and luciferase transfection assays were conducted according to the protocol of the Luciferase Assay system (Promega). The total protein amount was measured by BCA assay kit (Applygen Technologies Inc.). The Luciferase relative light unit (RLU) was analyzed to evaluate the cell transfection efficiency, and the results are expressed as mean and SD (*n* = 3).

### 2.11. Intracellular Localization of the Steroid-Based Cationic Lipids/Cy3-pDNA Lipoplexes Observed by Fluorescence Microscopy 

H1299 cells were seeded into 6-well microplates (4 × 10^5^ cells/well in 1 mL DMEM medium with 10% FBS), and incubated at 37 °C under 5% CO_2_ for 24 h. Then, the as-prepared steroid-based cationic lipids/Cy3-pDNA (± = 10) and Lipo2000/Cy3-pDNA (*w*/*w* = 3) were added into each well and incubated for 4 h. Before fluorescence imaging, H1299 cells were washed with 1 × PBS three times to diminish the fluorescence background. Then, the cells were fixed with 4% paraformaldehyde in 0.12 M phosphate buffer (pH 7.2) for 10 min, and washed three times with 1 × PBS. Thereafter, DAPI (for cell nuclei staining, 0.5 µg/per well) and Lysotracker (for lysosome staining, 2.5 µg/per well) were added and incubated for 15 min to stain the cell nuclei and lysosome. Finally, the H1299 cells were washed with 1 × PBS three times and the fluorescent images were recorded on a Nikon Ti-S invert fluorescence microscopy. 

## 3. Results and Discussion

### 3.1. Synthesis and Characterization of the Steroid-Based Cationic Lipids

**Scheme 1:**
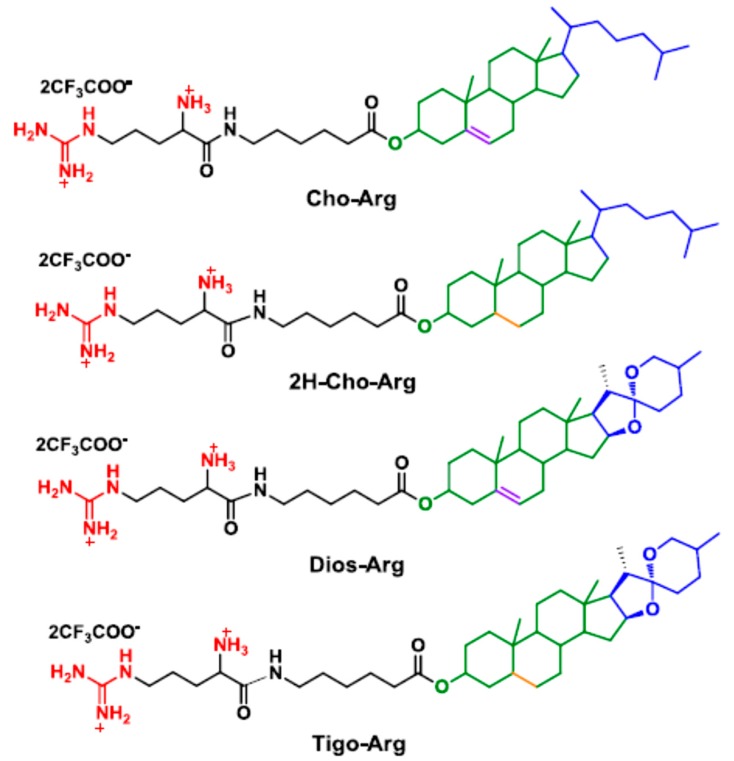
Molecular structures of the steroid-based cationic lipids.

Generally, in cationic lipid-based gene carriers, cationic headgroups are connected to hydrophobic lipid tails via certain linkers [39]. As a modular synthetic approach, herein, we prepared four steroid-based cationic lipid analogs (Scheme 1) by facile coupling of the steroid hydrophobes/skeletons (cholesterol, 2H-cholesterol, diosgenin and tigogenin) with Boc-protected (*l*-)-arginine headgroup through 6-aminocaproic acid, a natural metabolite of (*l*-)-Lysine. The synthetic procedures and routes are depicted in S1 and Scheme S1 in the Supplementary Materials. Firstly, steroid hydrophobes were reacted with 6-(Boc)-aminocaproic acid with the catalysis of DCC/DMAP [25] to prepare the steroid-caproic lipid precursors (^1^H NMR spectra shown in Figure S1a). Then, the as-prepared precursors were further coupled with Boc-protected (*l*-)-arginine (Boc-Arg-OH) in the presence of EDC/DMAP to synthesis the Boc-protected steroid-arginine cationic lipid precursors. Finally, the BOC protection group was removed in excessive trifluoroacetic acid [40] to afford the steroid-based cationic lipids (Cho-Arg, 2H-Cho-Arg, Dios-Arg and Tigo-Arg) as products with the isolation yield of 52–63%. Their structural characterization by NMR and ESI-MS are described in detail in the Experimental Section (Supplementary Materials S1), and the ^1^H NMR spectrum of the lipids are shown in Figure S1b. Proton signals at around 7.7, 8.2 and 8.5 ppm were identified as the protonated guanidino groups on the (*l*-)-arginine. The broad signal around 6.9–7.6 ppm belongs to the protonated α-primary amino group. Cho-Arg and Dios-Arg both showed obvious double bond proton signals at around 5.2–5.3 ppm, which could not be observed on 2H-Cho-Arg and Tigo-Arg lipids due to their hydrogen-saturated steroid skeletons. The results indicated the new steroid-based cationic lipids were successfully prepared with facile and efficient synthetic approaches.

### 3.2. pDNA Binding of the Steroid-Based Cationic Lipids and the Stability of the Lipoplexes

**Figure 1:**
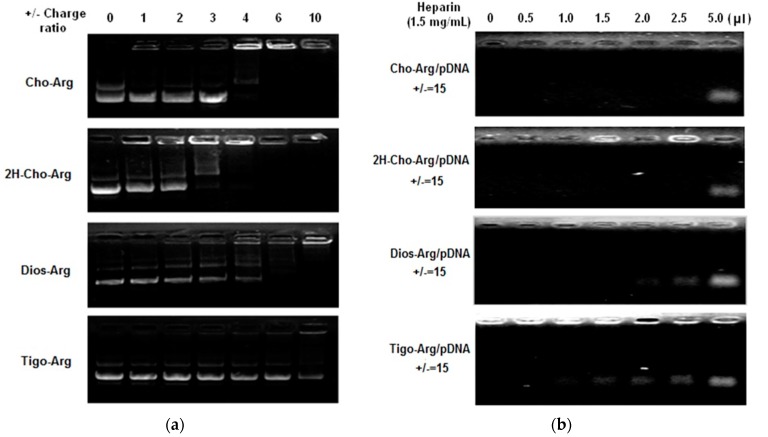
(**a**) Agarose-gel retardant assay of pDNA binding affinity for the steroid-based cationic lipids under various +/− charge ratios; (**b**) Heparin competition assay of the stability of the steroid-based cationic lipids/pDNA lipoplexes (± = 15).

Highly efficient lipid gene carriers should bind/load plasmid DNA at low +/− charge ratios [38]. Herein, pDNA binding affinities of the steroid-based cationic lipids were measured by agarose gel DNA-retardation assay [41]. As shown in Figure 1a, in the presence of 10 mM PBS in TAE buffer solution, the intensity of the migrating free pDNA bands decreases gradually with the increasing amount of steroid-based cationic lipids. Cho-Arg and 2H-Cho-Arg showed higher pDNA binding affinities that could completely retard pDNA at +/− ratio of 4, while Dios-Arg and Tigo-Arg have lower pDNA binding affinity (± > 6). The pDNA binding affinity of the steroid-based cationic lipids was in a sequence of: Cho-Arg (± = 4) ≈ 2H-Cho-Arg (± = 4) > Dios-Arg (± = 6) > Tigo-Arg (± >10), eliciting the pDNA binding affinity was steroid-skeleton dependent. We had revealed that pDNA binding affinity of some cholesterol-based cationic lipids relied largely on the pKa values of headgroups [25]; the above-mentioned results indicated that the pDNA binding affinity could be tuned by choosing certain types of steroid hydrophobes/skeletons for the construction of cationic lipids.

For practical applications, the pDNA-loaded lipoplexes should be stable enough to avoid the possible dissociation in physiological conditions before they reached targeting cells/tissues. The stability of steroid-based cationic lipids/pDNA lipoplexes were evaluated by heparin competition assay according to the literature [42]. As shown in Figure 1b, the pDNA migration band appeared from the lipoplexes after addition of different amount of heparin (1.5 mg/mL), due to the heparin induced dissociation of lipoplexes. The lipoplexes stability was in a sequence of: Cho-Arg/pDNA ≈ 2H-Cho-Arg/pDNA > Dios-Arg/pDNA > Tigo-Arg/pDNA. Notably, the trends of lipoplexes stability by heparin competition assays were in good agreement with the results of pDNA binding affinity, indicating that the introduction of flexible hydrophobic tails [43] could enhance the lipoplex stabilities. Moreover, the stability differences among the lipoplexes may bring them different gene transfection efficiencies.

### 3.3. Particle Size, Surface Charge and Morphology of the Lipoplexes

Prior works revealed that gene transfection and cellular trafficking manners of lipoplex nanoparticles relied greatly on their size, shape, and surface charge [44], which depended on structural factors such as cationic headgroups, lipid hydrophobes, etc. [45]. Herein, the hydrodynamic particle size and surface charge of the steroid-based cationic lipids/pDNA lipoplexes were measured by DLS. As shown in Figure S2a, the lipoplexes showed different average particle sizes. Cho-Arg and 2H-Cho-Arg formed smaller lipoplexes (92–110 nm), whereas much bigger particle sizes were observed on Dios-Arg (190–205 nm) and Tigo-Arg (318–390 nm) lipoplexes, indicating the steroid hydrophobes/skeletons played important roles in lipoplexes formation. Meanwhile, different lipoplex surface charges were observed (Figure S2b). Cho-Arg/pDNA and 2H-Cho-Arg/pDNA lipoplexes demonstrated a relatively rapid surface charge increasing from −18–−15 mV to +28–+36 mV when the +/− ratio increasing from 2 to 30, whilst Dios-Arg/pDNA and Tigo-Arg/pDNA showed comparatively slower charge increasing (from −18–−15 mV to +23–+28 mV) within the same +/− range, elucidating that the surface charge could be tuned by selection of particular steroid skeletons. Moreover, the steroid-lipids/pDNA lipoplex nanoparticles demonstrated various size distributions at the +/− ratio of 15: Cho-Arg/pDNA and 2H-Cho-Arg/pDNA lipoplexes have lower size dispersity (PDI < 0.09), whereas Dios-Arg/pDNA showed higher size dispersity (PDI = 0.15) and Tigo-Arg/pDNA demonstrate the highest dispersity (PDI = 0.25). The results suggested that the steroid-based cationic lipids/pDNA lipoplexes might possess different gene transfection efficiency and intracellular behaviors [25]. 

Morphologies of the steroid lipids/pDNA lipoplexes were further investigated by TEM. In Figure S2c, the steroid lipids/pDNA lipoplexes (± = 15) were observed as near-spherical nanoparticles [24]. Cho-Arg and 2H-Cho-Arg/pDNA lipoplexes were seen as small nanoparticles (40–110 nm) and Dios-Arg/pDNA were bigger nanoparticles (120–210 nm), whereas Tigo-Arg/pDNA showed the biggest particle sizes of 180–340 nm. Likewise, Yu et al. [46] recently reported that the lipoplexes particle size of a biotin-conjugated diosgenin cationic lipid were larger than their cholesterol-containing analogs. The trend of steroid-based lipids/pDNA lipoplexes morphology measured by TEM was in accordance with the DLS data. Compared to DLS results, smaller lipoplex particle size was observed by TEM, which could be interpreted as a result of nanoparticle shrinking in the drying process during TEM sample preparation [47].

It had been disclosed that the interaction of cationic gene carriers with serum proteins could affect their gene transfection properties [18]. To further examine the interaction between the lipoplexes and serum proteins, the average particle size of the steroid-based lipids/pDNA lipoplexes (± = 15) were measured in the presence of bovine serum albumin [48] (BSA, 1 mg/mL) after incubation for 20 min (Figure S2d). The average particle sizes of Dios-Arg/pDNA and Tigo-Arg/pDNA found largely significantly increased (49–57 nm), whereas Cho-Arg/pDNA and 2H-Cho-Arg/pDNA only showed slight particle size increases (8–15 nm). The results elicited the steroid-based cationic lipids may have different gene transfection properties under serum environments.

### 3.4. Cytotoxicity of the Steroid-Based Cationic Lipids by MTT/CCK-8 Assay

**Figure 2:**
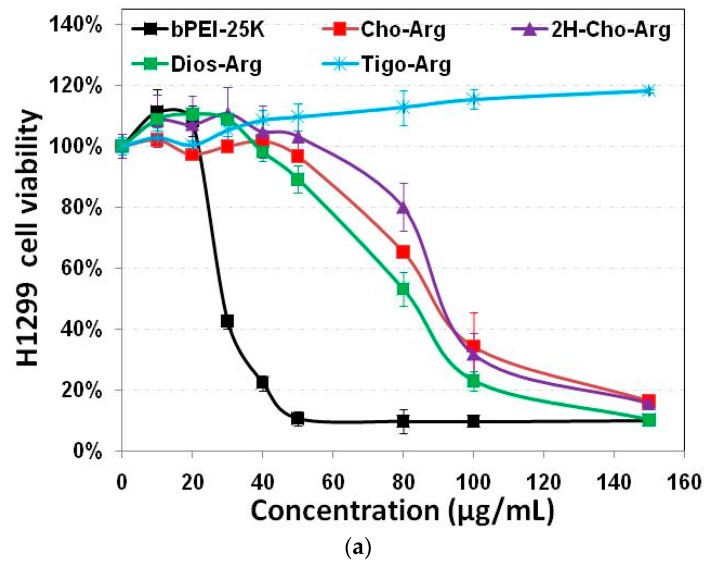

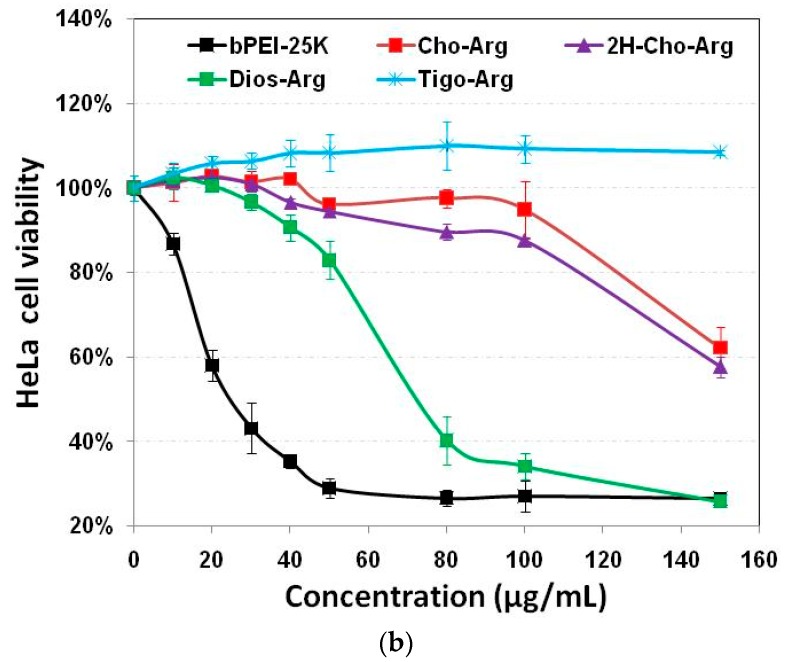
Cytotoxicity of the steroid-based cationic lipids with various concentrations (from 0 to 150 µg/mL) in: (**a**) H1299 cells; and (**b**) HeLa cells.

Developing highly biocompatible cationic gene carriers was essential for safe gene delivery/transfection. We evaluated the in vitro cytotoxicity of the steroid-based cationic lipids in H1299 and HeLa cells by MTT and CCK-8 assays with bPEI-25k as the control. As shown in Figure 2a,b, very high cytotoxicity of the bPEI-25k control was observed in both H1299 and HeLa cell lines (IC_50_ = 28.8 µg/mL in H1299; and IC_50_ = 23.7 µg/mL in HeLa), due to its strong cell membrane disruption effect [49]. Notably, the steroid-based lipids have different cytotoxicities within the concentration range from 0 to 150 µg/mL. Cho-Arg (IC_50_ = 88.5 µg/mL in H1299; and IC_50_ > 150.0 µg/mL in HeLa) and 2H-Cho-Arg (IC_50_ = 92.7 µg/mL in H1299; and IC_50_ > 150.0 µg/mL in HeLa) showed moderate cell viability. Dios-Arg showed much higher cytotoxicity (IC_50_ = 83.5 µg/mL in H1299; and IC_50_ = 74.1 µg/mL in HeLa), which may be due to the inhibition effect of diosgenin on 3-hydroxy-3-methylglutaryl CoenzymeA (HMG-CoA) reductase, a rate-limiting enzyme involved in cholesterol biosynthesis [50]. Tigo-Arg showed much lower cytotoxicity than the other three lipids in H1299 and HeLa cells, but the mechanism is not clear yet. Similar trends of the cytotoxicity were also observed in COS-7 cell line (Figure S3). Interestingly, we noticed that there is only a double bond difference between the structures of Dios-Arg and Tigo-Arg, indicating the molecular structure (even a double bond) of the steroid hydrophobes could greatly affect the apparent cytotoxicity. Likewise, Yu et al. revealed that diosgenin-cyclen cationic lipid has a higher cytotoxicity than its cholesterol-cyclen counterpart [51]. In previous work, we have revealed that the apparent cytotoxicity of some cholesterol-based cationic lipids greatly relied on their cationic headgroups [25]. These results suggested that the apparent cytotoxicity could be controlled by selecting certain steroid hydrophobes/skeletons for the construction of cationic lipid gene carriers.

### 3.5. Luciferase Gene Transfection Assay of the Steroid-Based Cationic Lipids in the Absence/Presence of Serum

**Figure 3:**
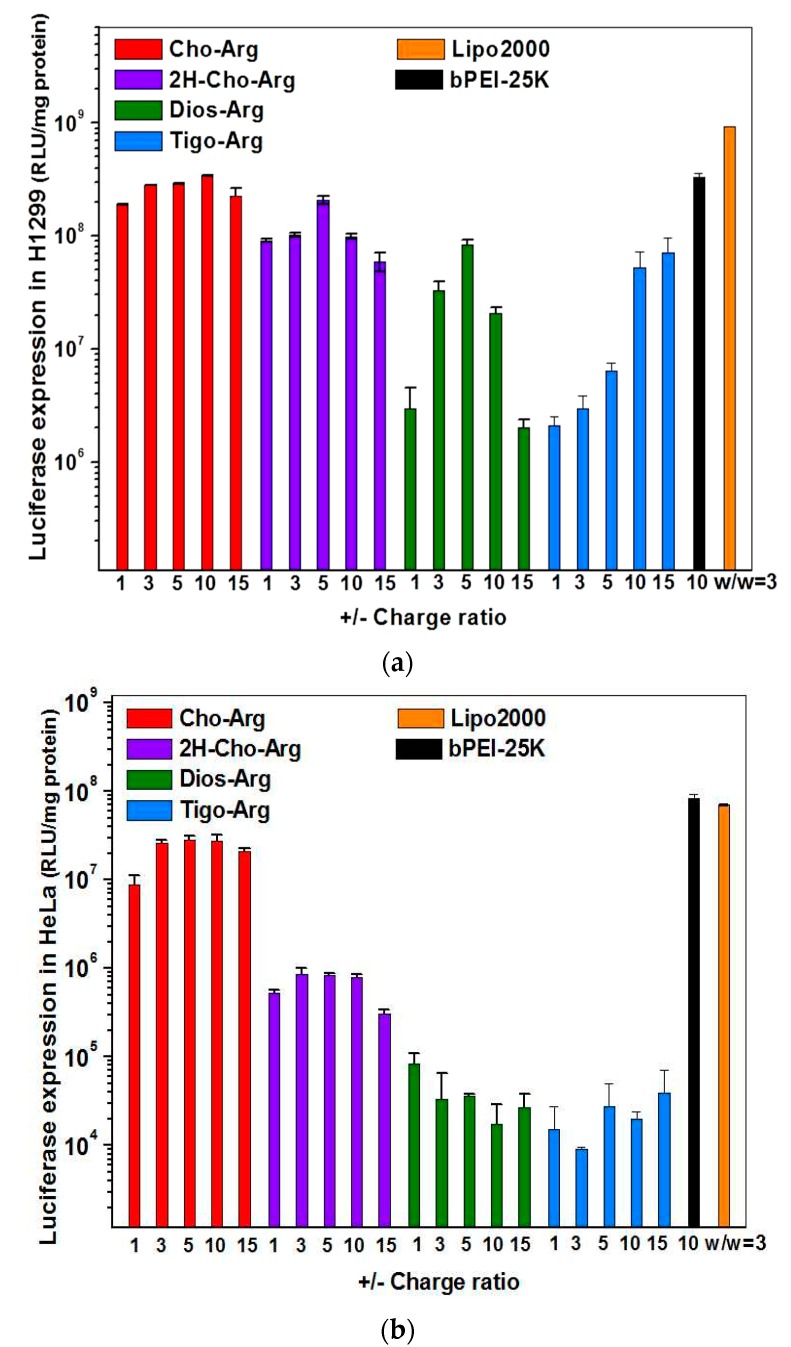
Luciferase gene transfection efficiency for the steroid-based cationic lipids/pDNA lipoplexes under various +/− charge ratios in: (**a**) H1299; and (**b**) HeLa cell lines. Commercially available bPEI-25K and Lipo2000 were used as controls.

Luciferase gene transfection efficiency of the steroid-based cationic lipids was evaluated in H1299 and HeLa cells in the presence/absence of serum [18]. The pDNA-loaded lipoplexes were firstly prepared by mixing the steroid-based cationic lipids with pDNA under various +/− charge ratios. Commercially available Lipo2000 and bPEI-25k were used as controls. As shown in Figure 3a,b, Cho-Arg showed the highest luciferase gene transfection capability (maximum: 3.5 × 10^8^ RLU in H1299; and 2.6 × 10^7^ RLU in HeLa, ± = 5–10), 1.5–87-fold greater than that of its non-double bond analog 2H-Cho-Arg (maximum: 2.0 × 10^8^ RLU in H1299; and 2.9 × 10^5^ RLU in HeLa cells). Meanwhile, Dios-Arg (maximum: 8.5 × 10^7^ RLU in H1299; and 9.3 × 10^4^ RLU in HeLa cells) and Tigo-Arg (7.1 × 10^7^ RLU in H1299; and 4.2 × 10^4^ RLU in HeLa cells) showed much lower transfection capability. Similar trends were also observed in pEGFP gene transfection assays (Figure S4). The results indicated that the steroid hydrophobes/skeletons played key roles in determining the gene transfection efficiency. Moreover, it can be noted that all the steroid lipids showed low cytotoxicity (>91.1% cell viability) within the pDNA transfection dose range (about 1.4–29.2 µg/mL, for lipoplexes ± 1–20) in H1299 and HeLa cell lines, indicating the steroid-based cationic lipids could be employed as comparatively safe carrier biomaterials for gene delivery application.

**Figure 4:**
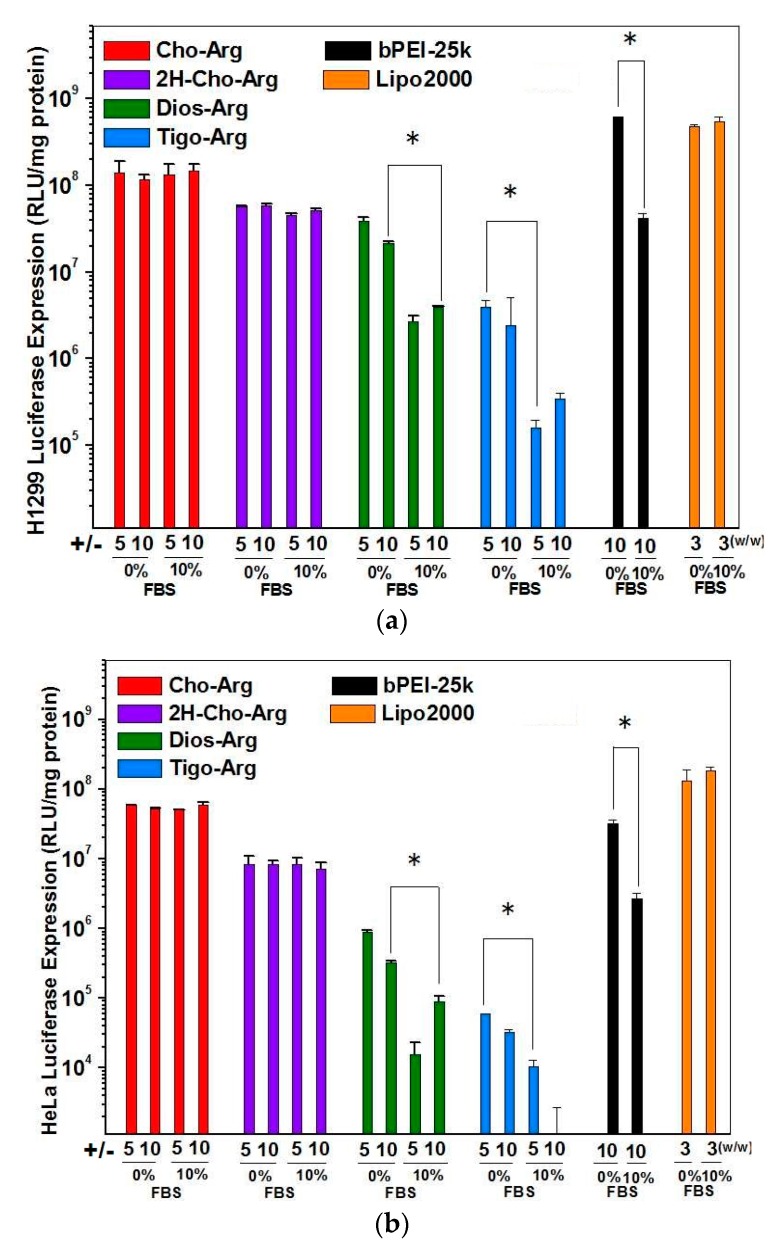
Luciferase gene transfection efficiency for the steroid-based cationic lipids/pDNA lipoplexes under various +/− charge ratios in the absence/presence of serum in: (**a**) H1299; and (**b**) HeLa cell lines. bPEI-25K and Lipo2000 were used as controls.

**Figure 5:**
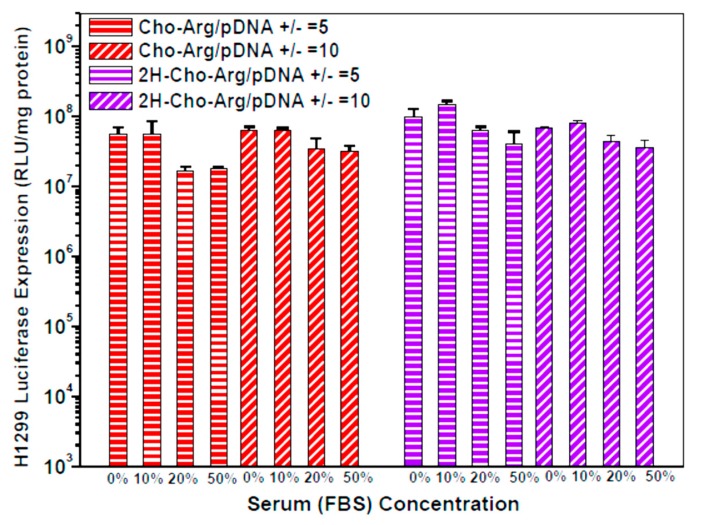
Luciferase gene transfection efficiency for the steroid-based cationic lipids/pDNA lipoplexes under +/− charge ratios of 5 and 10 in the presence of various concentrations of serum in H1299 cell lines.

For practical application, we further evaluated the Luciferase gene transfection efficiency in the presence of fetal bovine serum (10% FBS) [52]. As shown in Figure 4a,b, the luciferase gene transfection efficiency of Dios-Arg, Tigo-Arg and bPEI-25K drastically decreased in the presence of 10% FBS, while Cho-Arg, 2H-Cho-Arg and Lipo2000 do not show obvious decreasing of the gene transfection efficiency. Moreover, Cho-Arg and 2H-Cho-Arg could maintain high transfection efficiency even under higher serum concentrations (20–50% FBS, Figure 5). In the aforementioned results, Cho-Arg and 2H-Cho-Arg lipoplexes showed higher stability and smaller nanoparticle sizes than their Dios-Arg and Tigo-Arg counterparts, which may lead to their higher transfection efficiency. Moreover, Cho-Arg and 2H-Cho-Arg possess great serum-compatibility, similar to that of the multi-component transfection agent Lipo2000 [53] and surface “shielded” cationic polysulfobetaine PDMAEMA copolymers [54]. The results suggested that highly serum-compatible gene transfection could be achieved on single-component lipid gene carriers through molecular engineering approach. Notably, the optimized transfection efficiency of Cho-Arg was much higher than that of bPEI-25k and reached 1/3 of Lipo2000, which make it a potential gene transfection agent for practical application.

### 3.6. Cellular Uptake of the Steroid-Based Cationic Lipids/Cy3-pDNA Lipoplexes

**Figure 6:**
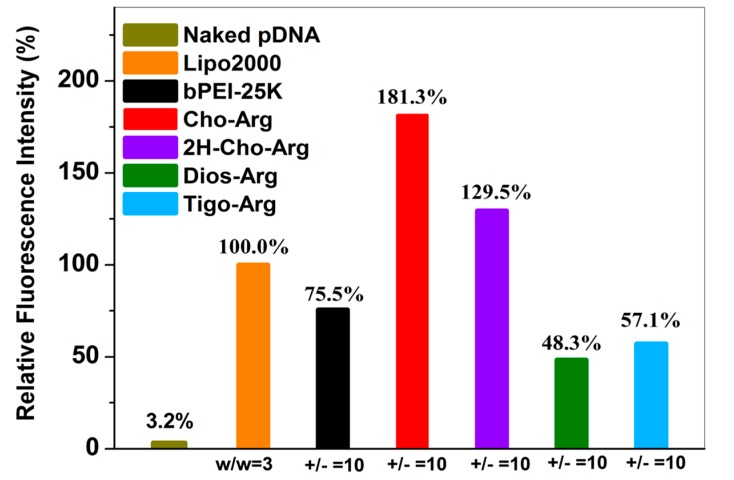
Intracellular uptake of the steroid-based cationic lipids/pDNA lipoplexes in HeLa cells analyzed by FACS. Lipo2000 and bPEI-25k were utilized as the controls for gene transfection in the optimized dose.

Cellular uptake capability was regarded as an essential factor that determines relevant gene transfection efficiency [55]. Herein, Cy3-pDNA was prepared by labeling pDNA with a red-fluorescent agent Cy3 using a Label IT^®^ Tracker™ Cy3-labeling kits [24]. Then, the steroid-based cationic lipids were incubated with Cy3-pDNA to prepare steroid-lipids/Cy3-pDNA lipoplexes. Cellular uptake of the lipoplexes (under the +/− ratio of 10) was analyzed by fluorescence microscope and flow cytometry. As shown in Figure S5a, strong red fluorescence emission (Cy3-DNA) were clearly observed inside the cells after 4 h incubation with the Cho-Arg/Cy3-pDNA, 2H-Cho-Arg/Cy3-pDNA and Lipo2000/Cy3-pDNA lipoplexes, while weaker red fluorescence emission were observed with Dios-Arg and Tigo-Arg/Cy3-pDNA lipoplexes, indicating the Cho-Arg and 2H-Cho-Arg lipoplexes possess higher cellular uptake capability than Dios-Arg and Tigo-Arg lipoplexes. Similar trends were also observed in H1299 cells after 24 h incubation (Figure S5b). Furthermore, quantitative analysis of the cellular uptake capability was conducted by flow cytometry (original profiles shown in Figure S6). As shown in Figure 6, under the +/− ratio of 10, compared to the bPEI-25K/Cy3-pDNA (75.5%) and Lipo2000/Cy3-pDNA (100.0%) controls, much higher uptake capability was observed for Cho-Arg/Cy3-pDNA (181.3%) and 2H-Cho-Arg/Cy3-pDNA (129.5%) lipoplexes, whereas Dios-Arg/Cy3-pDNA (48.3%) and Tigo-Arg/Cy3-pDNA (57.1%) lipoplexes showed lower cellular uptake capability. The results suggested that cellular uptake depends greatly on the steroid hydrophobes/skeletons. In addition, the high cellular uptake capability of Cho-Arg and 2H-Cho-Arg/pDNA lipoplexes may bring them high gene transfection efficiency [55]. 

### 3.7. Endocytosis Pathway Analysis of the Steroid-Based Cationic Lipids

**Figure 7:**
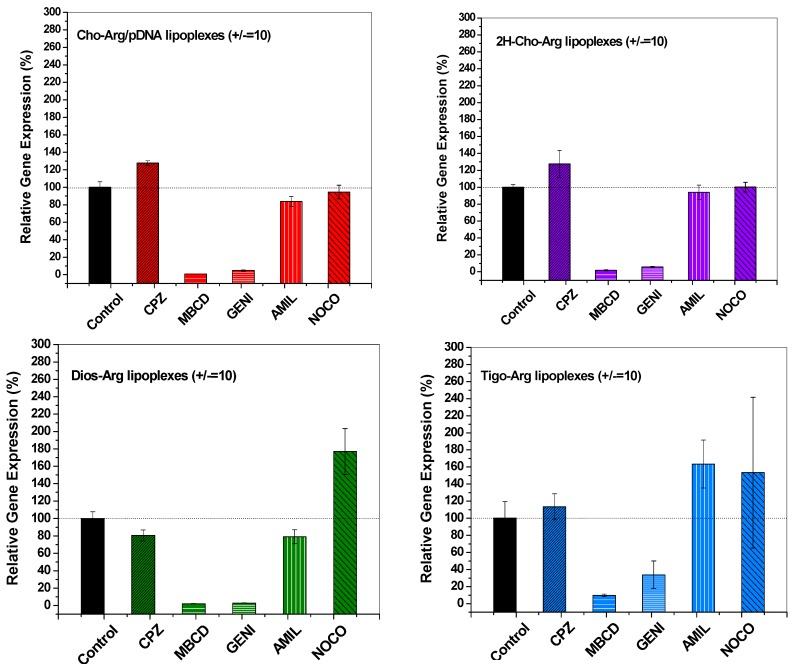
Endocytosis pathway analysis of the steroid-based cationic lipids/pDNA lipoplexes (± = 10) in H1299 cells by measuring the relative luciferase gene expression in the presence of different endocytosis-specific inhibitors: Chloropromazine (CPZ, clathrin-mediated endocytosis inhibitor); Methyl-β-cyclodextrin (MBCD, lipid-raft mediated endocytosis inhibitor); Genistein (GENI, caveolae mediated endocytosis inhibitor); Amiloride (AMIL, macropinocytosis mediated endocytosis inhibitor); and Nocodazole (NOCO, microtubule and actin inhibitor).

It had been revealed that the gene carriers/pDNA complexes entered the cell mainly through several endocytosis pathways, including clathrin-mediated endocytosis (inhibitor: Chlorpromazine, CPZ), caveloae-mediated endocytosis (inhibitor: Genistein, GENI), lipid-raft-mediated endocytosis (inhibitor: methyl-β-cyclodextrin, MBCD), micropinocytosis (inhibitor: Amiloride, AMIL) and microtubule-assisted phagocytosis (inhibitor: Nocodazole, NOCO). These pathways are directly relevant to the gene transfection efficiency [32,56,57]. To understand the endocytosis mechanisms of the steroid-based cationic lipids/pDNA lipoplexes, we analyzed the luciferase transfection in the presence of various endocytosis inhibitors, and the luciferase gene expression without addition of endocytosis inhibitors were set as 100%. As shown in Figure 7, the relative luciferase gene expression drastically decreased to 3.3–9.2% in the presence of lipid-raft mediated endocytosis inhibitor methyl-β-cyclodextrin (MBCD) [58], and also decreased to around 3.1–37.8% with the addition of caveolae-mediated endocytosis inhibitor genistein (GENI) [38], whilst the relative luciferase gene expression did not show dramatic decline in the presence of chlorpromazine (clathrin mediated endocytosis inhibitor) [59], amiloride (macropinocytosis inhibitor) [60] and nocodazole (microtubule inhibitor) [61], indicating the steroid-based cationic lipids/pDNA lipoplexes were mainly taken up by H1299 cells via caveolae and lipid-raft mediated pathways, which are regarded as cholesterol-dependent [10,62]. Notably, all the steroid-based cationic lipids/pDNA lipoplexes entered the H1299 cells via similar cholesterol-dependent endocytosis pathways, which suggests that changing the steroid hydrophobes/skeletons in the cationic lipids would not significantly alter the endocytosis pathway of their lipoplexes.

### 3.8. Intracellular Localization of the Steroid-Based Cationic Lipids

**Figure 8:**
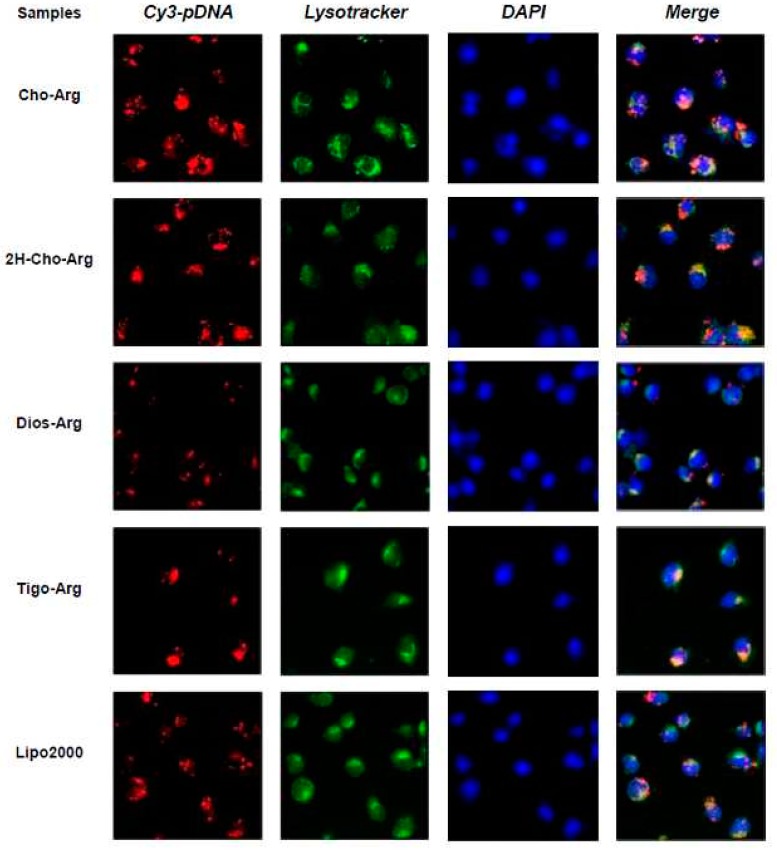
Fluorescence images (400×) for intracellular localization of the steroid-based cationic lipids/Cy3-pDNA lipoplexes after 4 h incubation in H1299 cells. Lipofectamine2000 (Lipo2000) was used as a reference. Red: Cy3-labeled pDNA; green: Lysotracker stained endosome/lysosome; blue: DAPI stained cell nuclei.

To get more insight into the cellular fate, the intracellular localization of the Cy3-pDNA-loaded lipoplexes in H1299 cells were observed by fluorescence microscope, with Lipo2000/Cy3-pDNA as the control. The cell nuclei and lysosome were separately labeled with fluorescent dyes DAPI (blue) and Lysotracker (green). As shown in Figure 8, after 4 h transfection with Cho-Arg/Cy3-pDNA and 2H-Cho-Arg/pDNA lipoplexes, strong red fluorescent spots were found within or around the DAPI-stained nucleus and part of them colocalized with the lysotracker-stained lysosome; Lipo2000/Cy3-pDNA lipoplexes showed similar localization effects. Meanwhile, with the transfection of Dios-Arg/Cy3-pDNA and Tigo-Arg/Cy3-pDNA lipoplexes, comparatively weaker red fluorescent spots were observed dispersed around or within the cell nuclei and lysosome, indicating their low cellular uptake capability, which may result in their low gene transfection efficiency. In prior works, we disclosed the lipoplexes of some cholesterol-disulfide cationic lipids (CHOSS) possess perinucleic localization effect [24], which may be due to the cholesterol-induced perinucleic localization. Moreover, we revealed that some (*l*-)-arginine-rich peptide mimics could enable the nuclei-oriented pDNA localization [38]. Likewise, Ma et al. showed that the “proton sponge” feature of (*l*-)-arginine could lead to lysosome localization effect [63]. Thus, in this case, the perinucleic, nucleic and lysosome localization effects of the steroid-based cationic lipids/Cy3-pDNA lipoplexes may be inferred as the synergistic contributions of the steroid hydrophobes/skeletons and (*l*-)-arginine headgroups. Accordingly, we proposed that an intracellular trafficking route of “lipid-raft-mediated endocytosis→lysosome→cell nucleic localization” may be involved in the intracellular trafficking of the lipoplexes. The results suggest that molecular structural factors including steroid hydrophobes/skeletons and (*l*-)-arginine headgroups could remarkably affect the intracellular localization of the pDNA-loaded lipoplexes, which also elicited a possible way to control the intracellular behaviors/mechanisms of the lipid gene delivery systems via molecular engineering approaches.

## 4. Conclusions

In summary, we successfully prepared a series of new steroid-based cationic lipids by incorporation of modular steroid hydrophobes and (*l*-)-arginine headgroup via facile and efficient synthetic approach. The study on the physico-chemical properties showed that the pDNA binding affinity of the steroid-based cationic lipids, average particle size, ζ potential, morphology and serum stability of the steroid-lipids/pDNA lipoplexes greatly depend on the steroid hydrophobes/skeletons. On the other hand, the biological features such as cytotoxicity, gene transfection efficiency, serum-compatibility and cellular uptake capability also strongly relied on the steroid hydrophobes/skeletons. Among the lipids, Cho-Arg showed remarkably high optimized pDNA transfection efficacy, even under the presence of 50% FBS, making it a latent transfection reagent for in vivo gene transfection. Interestingly, the steroid lipids/pDNA lipoplexes inclined to enter H1299 cells mainly through lipid-raft-mediated endocytosis pathway, following proposed intracellular trafficking routes of “lipid-raft-mediated endocytosis→lysosome escape→cell nucleic localization”. Compared to our previous works [24,25,31], this study provides deeper understanding of the structure–function relationships for the steroid-based cationic lipid gene carriers, especially the correlation between the lipid skeletons/structures and their endocytosis pathways and intracellular trafficking/localization, which may offer a new route to further design “endocytosis pathway maintainable/adjustable lipid nanomaterials”. Moreover, it also suggests that high-performance steroid-based lipid gene carriers could be achieved by optimizing the steroid hydrophobes/skeletons through molecular engineering approaches.

## Supplementary Materials

The following are available online at http://www.mdpi.com/1422-0067/19/2/369/s1.

## References

[1] Albrecht M.A., Evans C.W., Raston C.L. (2006). Green chemistry and the health implications of nanoparticles. Green Chem..

[2] Zhang K., Wang Y., Yu A., Zhang Y., Tang H., Zhu X.X. (2010). Cholic acid-modified dendritic multimolecular micelles and enhancement of anticancer drug therapeutic efficacy. Bioconjug. Chem..

[3] Li C., Lavigueur C., Zhu X.X. (2011). Aggregation and thermoresponsive properties of new star block copolymers with a cholic acid core. Langmuir.

[4] Guo X., Szoka F.C. (2003). Chemical approaches to triggerable lipid vesicles for drug and gene delivery. Acc. Chem. Res..

[5] Niidome T., Huang L. (2002). Gene therapy progress and prospects: Nonviral vectors. Gene Ther..

[6] Lv H., Zhang S., Wang B., Cui S., Yan J. (2006). Toxicity of cationic lipids and cationic polymers in gene delivery. J. Control. Release.

[7] Morille M., Passirani C., Vonarbourg A., Clavreul A., Benoit J.P. (2008). Progress in developing cationic vectors for non-viral systemic gene therapy against cancer. Biomaterials.

[8] Lee A.L.Z., Venkataraman S., Sirat S.B.M., Gao S., Hedrick J.L., Yang Y.Y. (2012). The use of cholesterol-containing biodegradable block copolymers to exploit hydrophobic interactions for the delivery of anticancer drugs. Biomaterials.

[9] Dao K.L., Hanson R.N. (2012). Targeting the estrogen receptor using steroid–therapeutic drug conjugates (hybrids). Bioconjug. Chem..

[10] Chen C.J., Wang J.C., Zhao E.Y., Gao L.Y., Feng Q., Liu X.Y., Zhao Z.X., Ma X.F., Hou W.J., Zhang L.R. (2013). Self-assembly cationic nanoparticles based on cholesterol-grafted bioreducible poly(amidoamine) for sirna delivery. Biomaterials.

[11] He Z.Y., Chu B.Y., Wei X.W., Li J., Carl E.K., Song X.R., He G., Xie Y.M., Wei Y.Q., Qian Z.Y. (2014). Recent development of poly(ethylene glycol)-cholesterol conjugates as drug delivery systems. Int. J. Pharm..

[12] Bhattacharya S., Bajaj A. (2009). Advances in gene delivery through molecular design of cationic lipids. Chem. Commun..

[13] Biswas J., Bajaj A., Bhattacharya S. (2010). Membranes of cationic gemini lipids based on cholesterol with hydroxyl headgroups and their interactions with DNA and phospholipid. J. Phys. Chem. B.

[14] Bajaj A., Kondaiah P., Bhattacharya S. (2008). Effect of the nature of the spacer on gene transfer efficacies of novel thiocholesterol derived gemini lipids in different cell lines: A structure–activity investigation. J. Med. Chem..

[15] Bajaj A., Kondaiah P., Bhattacharya S. (2008). Synthesis and gene transfection efficacies of pei−cholesterol-based lipopolymers. Bioconjug. Chem..

[16] Bajaj A., Kondiah P., Bhattacharya S. (2007). Design, synthesis, and in vitro gene delivery efficacies of novel cholesterol-based gemini cationic lipids and their serum compatibility: A structure-activity investigation. J. Med. Chem..

[17] Bajaj A., Kondaiah P., Bhattacharya S. (2007). Synthesis and gene transfer activities of novel serum compatible cholesterol-based gemini lipids possessing oxyethylene-type spacers. Bioconjug. Chem..

[18] Misra S.K., Naz S., Kondaiah P., Bhattacharya S. (2014). A cationic cholesterol based nanocarrier for the delivery of p53-egfp-c3 plasmid to cancer cells. Biomaterials.

[19] Ghosh A., Mukherjee K., Jiang X., Zhou Y., McCarroll J., Qu J., Swain P.M., Baigude H., Rana T.M. (2010). Design and assembly of new nonviral rnai delivery agents by microwave-assisted quaternization (maq) of tertiary amines. Bioconjug. Chem..

[20] Medvedeva D.A., Maslov M.A., Serikov R.N., Morozova N.G., Serebrenikova G.A., Sheglov D.V., Latyshev A.V., Vlassov V.V., Zenkova M.A. (2009). Novel cholesterol-based cationic lipids for gene delivery. J. Med. Chem..

[21] Ivanova E.A., Maslov M.A., Kabilova T.O., Puchkov P.A., Alekseeva A.S., Boldyrev I.A., Vlassov V.V., Serebrennikova G.A., Morozova N.G., Zenkova M.A. (2013). Structure-transfection activity relationships in a series of novel cationic lipids with heterocyclic head-groups. Org. Biomol. Chem..

[22] Maslov M.A., Kabilova T.O., Petukhov I.A., Morozova N.G., Serebrennikova G.A., Vlassov V.V., Zenkova M.A. (2012). Novel cholesterol spermine conjugates provide efficient cellular delivery of plasmid DNA and small interfering rna. J. Control. Release.

[23] Maslov M.A., Morozova N.G., Chizhik E.I., Rapoport D.A., Ryabchikova E.I., Zenkova M.A., Serebrennikova G.A. (2010). Synthesis and delivery activity of new cationic cholesteryl glucosides. Carbohydr. Res..

[24] Sheng R., Luo T., Zhu Y., Li H., Sun J., Chen S., Sun W., Cao A. (2011). The intracellular plasmid DNA localization of cationic reducible cholesterol-disulfide lipids. Biomaterials.

[25] Sheng R., Luo T., Li H., Sun J., Wang Z., Cao A. (2014). Cholesterol-based cationic lipids for gene delivery: Contribution of molecular structure factors to physico-chemical and biological properties. Colloids Surf. B.

[26] Cline L.L., Janout V., Fisher M., Juliano R.L., Regen S.L. (2011). A molecular umbrella approach to the intracellular delivery of small interfering rna. Bioconjug. Chem..

[27] Janout V., Cline L.L., Feuston B.P., Klein L., O’Brien A., Tucker T., Yuan Y., O’Neill-Davis L.A., Peiffer R.L., Nerurkar S.S. (2014). Molecular umbrella conjugate for the ocular delivery of sirna. Bioconjug. Chem..

[28] Yi W.J., Zhang Q.F., Zhang J., Liu Q., Ren L., Chen Q.M., Guo L., Yu X.Q. (2014). Cyclen-based lipidic oligomers as potential gene delivery vehicles. Acta Biomater..

[29] Zhang Q.F., Yi W.J., Wang B., Zhang J., Ren L., Chen Q.M., Guo L., Yu X.Q. (2013). Linear polycations by ring-opening polymerization as non-viral gene delivery vectors. Biomaterials.

[30] Liu J.L., Ma Q.P., Huang Q.D., Yang W.H., Zhang J., Wang J.Y., Zhu W., Yu X.Q. (2011). Cationic lipids containing protonated cyclen and different hydrophobic groups linked by uracil-pna monomer: Synthesis and application for gene delivery. Eur. J. Med. Chem..

[31] Sheng R., Luo T., Li H., Sun J., Wang Z., Cao A. (2013). “Click” synthesized sterol-based cationic lipids as gene carriers, and the effect of skeletons and headgroups on gene delivery. Bioorg. Med. Chem..

[32] Xiang S., Tong H., Shi Q., Fernandes J.C., Jin T., Dai K., Zhang X. (2012). Uptake mechanisms of non-viral gene delivery. J. Control. Release.

[33] Bae Y.U., Kim B.K., Park J.W., Seu Y.B., Doh K.O. (2012). Endocytic pathway and resistance to cholesterol depletion of cholesterol derived cationic lipids for gene delivery. Mol. Pharm..

[34] Pozzi D., Marchini C., Cardarelli F., Rossetta A., Colapicchioni V., Amici A., Montani M., Motta S., Brocca P., Cantù L. (2013). Mechanistic understanding of gene delivery mediated by highly efficient multicomponent envelope-type nanoparticle systems. Mol. Pharm..

[35] Nam H.Y., Kwon S.M., Chung H., Lee S.Y., Kwon S.H., Jeon H., Kim Y., Park J.H., Kim J., Her S. (2009). Cellular uptake mechanism and intracellular fate of hydrophobically modified glycol chitosan nanoparticles. J. Control. Release.

[36] Zhu C., Jung S., Luo S., Meng F., Zhu X., Park T.G., Zhong Z. (2010). Co-delivery of sirna and paclitaxel into cancer cells by biodegradable cationic micelles based on PDMAEMA–PCL–PDMAEMA triblock copolymers. Biomaterials.

[37] Li L., Song H., Luo K., He B., Nie Y., Yang Y., Wu Y., Gu Z. (2011). Gene transfer efficacies of serum-resistant amino acids-based cationic lipids: Dependence on headgroup, lipoplex stability and cellular uptake. Int. J. Pharm..

[38] Li H., Luo T., Sheng R., Sun J., Wang Z., Cao A. (2013). Endoplasmic reticulum localization of poly(ω-aminohexyl methacrylamide)s conjugated with (*l*-)-arginines in plasmid DNA delivery. Biomaterials.

[39] Zhi D., Zhang S., Cui S., Zhao Y., Wang Y., Zhao D. (2013). The headgroup evolution of cationic lipids for gene delivery. Bioconjug. Chem..

[40] Sheng R., An F., Wang Z., Li M., Cao A. (2015). Assembly of plasmid DNA with pyrene-amines cationic amphiphiles into nanoparticles and their visible lysosome localization. RSC Adv..

[41] Zhu Y., Sheng R., Luo T., Li H., Sun W., Li Y., Cao A. (2011). Amphiphilic cationic dendritic poly(l-lysine) -block-poly(l-lactide)-block-dendritic poly(l-lysine) s in aqueous solution: Self-aggregation and interaction with DNA as gene delivery carriers. Macromol. Biosci..

[42] Moret I., Esteban Peris J., Guillem V.M., Benet M., Revert F., Dasí F., Crespo A., Aliño S.F. (2001). Stability of PEI–DNA and DOTAP–DNA complexes: Effect of alkaline pH, heparin and serum. J. Control. Release.

[43] Sheikhi Mehrabadi F., Fischer W., Haag R. (2012). Dendritic and lipid-based carriers for gene/sirna delivery (a review). Curr. Opin. Solid Stat. Mater. Sci..

[44] Rehman Z.U., Zuhorn I.S., Hoekstra D. (2013). How cationic lipids transfer nucleic acids into cells and across cellular membranes: Recent advances. J. Control. Release.

[45] LaManna C.M., Lusic H., Camplo M., McIntosh T.J., Barthélémy P., Grinstaff M.W. (2012). Charge-reversal lipids, peptide-based lipids, and nucleoside-based lipids for gene delivery. Acc. Chem. Res..

[46] Liu Q., Yi W.J., Zhang Y.-M., Zhang J., Guo L., Yu X.Q. (2013). Biotinylated cyclen-contained cationic lipids as non-viral gene delivery vectors. Chem. Biol. Drug Des..

[47] Maggi F., Ciccarelli S., Diociaiuti M., Casciardi S., Masci G. (2011). Chitosan nanogels by template chemical cross-linking in polyion complex micelle nanoreactors. Biomacromolecules.

[48] Gan Q., Wang T., Cochrane C., McCarron P. (2005). Modulation of surface charge, particle size and morphological properties of chitosan–tpp nanoparticles intended for gene delivery. Colloids Surf. B.

[49] Kunath K., von Harpe A., Fischer D., Petersen H., Bickel U., Voigt K., Kissel T. (2003). Low-molecular-weight polyethylenimine as a non-viral vector for DNA delivery: Comparison of physicochemical properties, transfection efficiency and in vivo distribution with high-molecular-weight polyethylenimine. J. Control. Release.

[50] Raju J., Bird R.P. (2007). Diosgenin, a naturally occurring furostanol saponin suppresses 3-hydroxy-3-methylglutaryl coa reductase expression and induces apoptosis in hct-116 human colon carcinoma cells. Cancer Lett..

[51] Huang Q.-D., Ou W.-J., Chen H., Feng Z.H., Wang J.Y., Zhang J., Zhu W., Yu X.Q. (2011). Novel cationic lipids possessing protonated cyclen and imidazolium salt for gene delivery. Eur. J. Pharm. Biopharm..

[52] Sun Y.X., Yang B., Chen S., Lei Q., Feng J., Qiu X.F., Dong N.G., Zhuo R.X., Zhang X.Z. (2013). Oligoamines grafted hyperbranched polyether as high efficient and serum-tolerant gene vectors. Colloids Surf. B.

[53] Dalby B., Cates S., Harris A., Ohki E.C., Tilkins M.L., Price P.J., Ciccarone V.C. (2004). Advanced transfection with lipofectamine 2000 reagent: Primary neurons, sirna, and high-throughput applications. Methods.

[54] Dai F., Liu W. (2011). Enhanced gene transfection and serum stability of polyplexes by PDMAEMA-polysulfobetaine diblock copolymers. Biomaterials.

[55] Shan Y., Luo T., Peng C., Sheng R., Cao A., Cao X., Shen M., Guo R., Tomás H., Shi X. (2012). Gene delivery using dendrimer-entrapped gold nanoparticles as nonviral vectors. Biomaterials.

[56] Adler A.F., Leong K.W. (2010). Emerging links between surface nanotechnology and endocytosis: Impact on nonviral gene delivery. Nano Today.

[57] Sahay G., Alakhova D.Y., Kabanov A.V. (2010). Endocytosis of nanomedicines. J. Control. Release.

[58] Hsu C.Y.M., Uludağ H. (2012). Cellular uptake pathways of lipid-modified cationic polymers in gene delivery to primary cells. Biomaterials.

[59] Wang L.H., Rothberg K.G., Anderson R. (1993). Mis-assembly of clathrin lattices on endosomes reveals a regulatory switch for coated pit formation. J. Cell Biol..

[60] Koivusalo M., Welch C., Hayashi H., Scott C.C., Kim M., Alexander T., Touret N., Hahn K.M., Grinstein S. (2010). Amiloride inhibits macropinocytosis by lowering submembranous pH and preventing rac1 and cdc42 signaling. J. Cell Biol..

[61] Ondřej V., Lukášová E., Falk M., Kozubek S. (2007). The role of actin and microtubule network in plastid DNA intracellular trafficking. Acta Biochim. Pol..

[62] Zhang R., Zheng N., Song Z., Yin L., Cheng J. (2014). The effect of side-chain functionality and hydrophobicity on the gene delivery capabilities of cationic helical polypeptides. Biomaterials.

[63] Ma D., Lin Q.M., Zhang L.M., Liang Y.Y., Xue W. (2014). A star-shaped porphyrin-arginine functionalized poly (l-lysine) copolymer for photo-enhanced drug and gene co-delivery. Biomaterials.

