# Supplementary material for: Skeleton-Controlled pDNA Delivery of Renewable Steroid-Based Cationic Lipids, the Endocytosis Pathway Analysis and Intracellular Localization
Source: Int J Mol Sci. 2018 Jan 26;19(2):369. doi: 10.3390/ijms19020369 (PMC5855591; doi:10.3390/ijms19020369)
Supplement: Supplementary file 1 [file ijms-19-00369-s001.pdf]

Supplementary information

Skeleton-Controlled pDNA Delivery of Renewable Steroid-Based Cationic Lipids, the Endocytosis Pathway Analysis and Intracellular Localization

Ruilong Sheng [1,2,3,*], Zhao Wang [1], Ting Luo [1], Amin Cao [1,*], Jingjing Sun [1] and Joseph M. Kinsella [2,*]

S1. Synthesis procedures of the steroid-based lipids

Scheme S1. Synthesis routes of the steroid-based cationic lipids.

Figure S1a. ^{1}H NMR spectra of the important intermediates of steroid-based cationic lipids; **Figure S1b**. ^{1}H NMR spectra of the steroid-based cationic lipids in d_6-DMSO solution.

Figure S2. (**a**) Average particle size; and (**b**) zeta potential of the steroid-based cationic lipids/pDNA lipoplexes under various +/− ratios measured by DLS at 25°C (λ = 633 nm, scattering angle: 90°). (**c**) TEM images of the lipoplexes (± = 15) at dried state. (**d**) The average particle size of the lipoplexes (± = 15) under the presence of BSA (1mg/mL).

Figure S3. The cytotoxicity (MTT) assays of the steroid-based cationic lipids in various concentrations (from 0 to 200 μg/mL) in COS-7 cells.

Figure S4. EGFP expression of the H1299 cell transfected with steroid-based cationic lipids/pEGFP DNA lipoplexes (± = 10) observed; Lipo2000 was used as the control.

Figure S5. Fluorescence images (400×) of cellular uptake of the steroid-based cationic lipids /Cy3-pDNA lipoplexes incubated with the gene carriers for: 4 h (**a**); and 24 h (**b**) in H1299 cells; Lipo2000 was used as a control.

Figure S6. Original profiles of the intracellular uptake of the steroid-based cationic lipids/pDNA lipoplexes in HeLa cells analyzed by FACS, the Lipo2000 and bPEI-25k were utilized as the controls for gene transfection in the optimized dose.

S1. Synthesis Procedures of the Steroid-Based Lipids

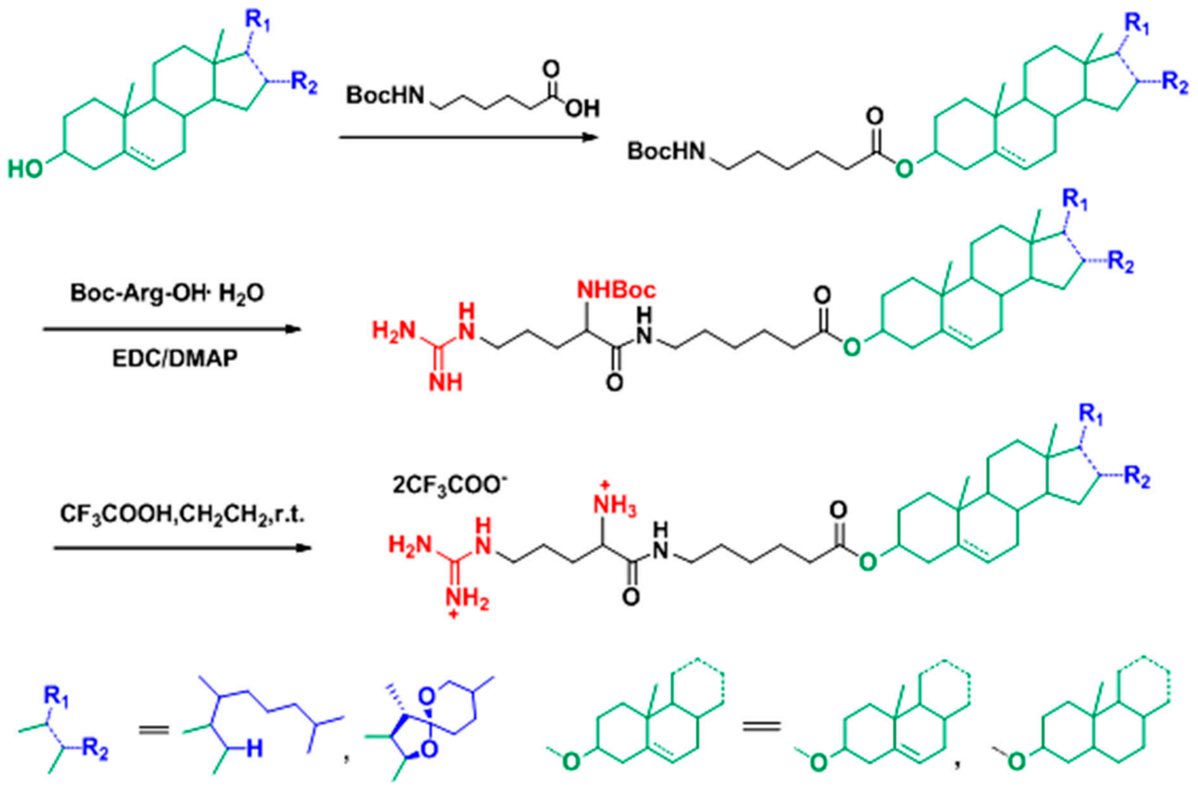

Scheme S1. Synthesis routes of the steroid-based cationic lipids.

1. General procedures of the synthesis of Steroid-Cap-Boc intermediates

To a 100 mL flask was added steroids (1.0 mmol), Boc-N-Caproic acid (1.5 mmol) and DCC/DMAP (1.5 mmol /0.1 g) were dissolved in 50 mL of dichloromethane, and the mixture was then stirred at ambient temperature for 24 h. Afterwards, the Dicyclohexylurea (DCU) solids were removed by filtration, and the residual solution was concentrated under reduced pressure, and purified by flash column chromatography with mixed solution of EtOAC/Hexane=1/3 *v*/*v* to achieve Steroid-Cap-BOC as white solids with a synthetic yield of 65–78%.

Cho-Cap-BOC:

^{1}H NMR ($CDCl_3$, 300Hz): δ 5.38 (1H, -CH=C, cholesterol), 3.71 (2H, COO-CH-), 3.11 (2H, Boc-NH-CH_2), 2.30 (2H, -CH_2COO-), 1.42 (9H，Boc), 2.23-0.74 (m, 42H, cholesterol),0.63 (s, 3H, -CH3).

2H-Cho-Cap-BOC:

^{1}H NMR ($CDCl_3$, 300Hz): δ 3.81 (2H, COO-CH-), 3.11 (2H, Boc-NH-CH_2), 2.30 (2H, -CH_2COO-), 1.42 (9H，Boc), 2.23-0.74 (m, 44H, 2H-cholesterol), 0.64 (s, 3H, -CH3).

Dios-Cap-Boc:

^{1}H NMR ($CDCl_3$, 300Hz): δ 5.35 (1H, -CH=C, diosgenin), 4.11(2H, O-CH_2-), 3.71 (2H, COO-CH-), 3.12 (2H, Boc-NH-CH_2), 2.30 (2H, -CH_2COO-), 1.42 (9H，Boc), 2.23-0.97 (m, H, diosgenin). 0.79 (3H, s, -CH3), 0.76 (3H, s, -CH3).

Tigo-Cap-Boc:

^{1}H NMR ($CDCl_3$, 300Hz): δ 4.11(2H, O-C$\underline{H}_2$-), 3.71 (2H, COO-C$\underline{H}$-), 3.12 (2H, Boc-NH-C$\underline{H}_2$), 2.30 (2H, -C$\underline{H}_2$COO-), 1.42 (9H， Boc), 2.23-0.97 (m, H, tigogenin). 0.79 (3H, s, -CH3), 0.76 (3H, s, -CH3).

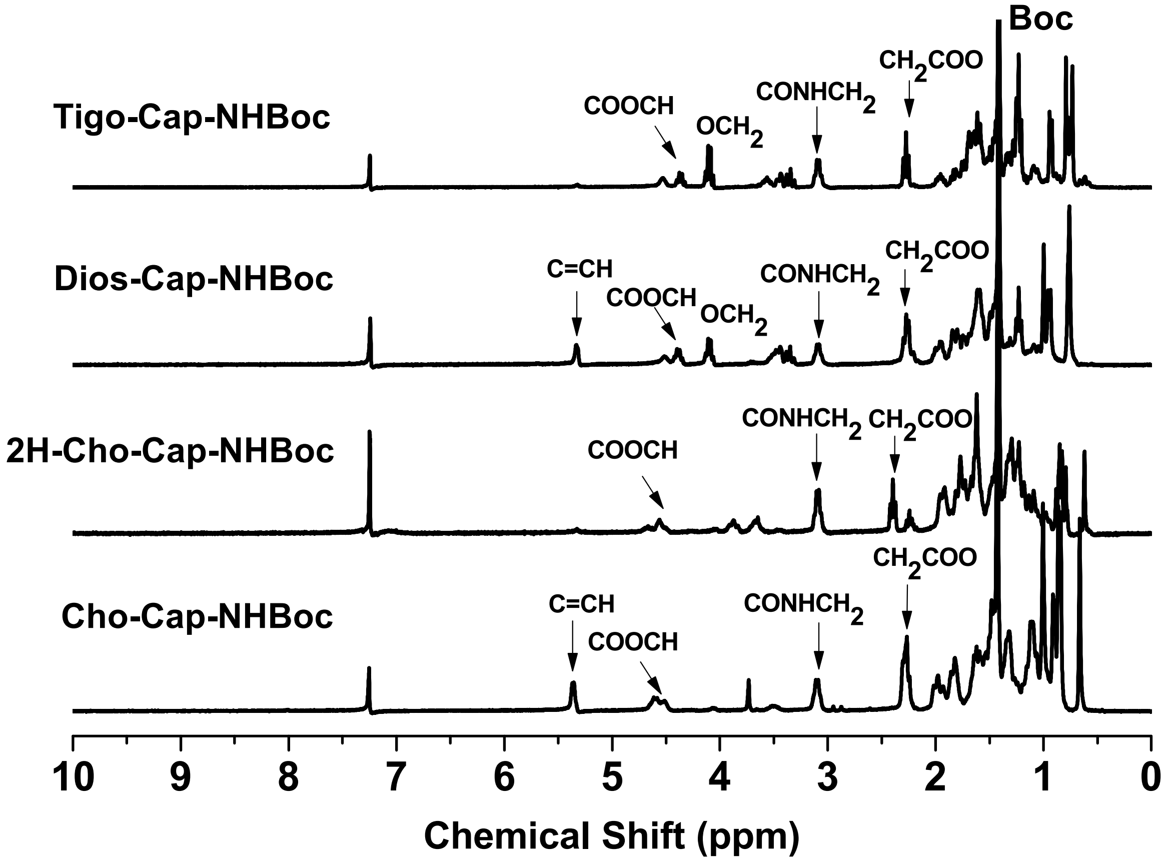

Figure S1a. ^{1}H NMR Spectra of the important intermediates of steroid-based cationic lipids.

2. General procedures of the synthesis of Steroid-based cationic lipids

To a 100 mL flask was added Steroid-Cap-BOC (0.5 mmol) dissolved in 20 mL of dichloromethane and 5 mL trifluoroacetic acid was added dropwise, the mixture was then stirred at ambient temperature for 5 h. Then, solvents were removed under reduced pressure to get Steroid-Cap-NH_2 as the intermediate without further purification. Then, the as-prepared Steroid-Cap-NH_2 intermediates, Arg-Boc·HCl·H_2O (0.75 mol), and EDC/DMAP (1.0 mmol/0.1 g) were dissolved in 50 mL dichloromethane with 5 mL triethylamine, and the mixture was stirred at ambient temperature for 24 h. Then, the solids were removed by filtration, and the filtrates were concentrated under reduced pressure and purified by flash column chromatography with mixed solution of EtOAC/ Methanol=10/1 (*v*/*v*) to get Boc-protected steroid-arginine conjugated lipids as yellowish solids. After that, the Boc protection groups were removed in 10 mL dichloromethane/trifluoroacetic acid (5 mL/5 mL) mixed solution and stirred for another 5 h, and then the solvent was removed and precipitated with diethyl ether. The steroid-arginine conjugated lipids were obtained as final product with a total synthetic yield of 52–63%.

Cho-Arg:

^{1}H NMR ($CDCl_3$, 300Hz): δ 7.70-8.50 (Guanidyl-H, arginine), 7.0-7.60 (H, NH_3^+), 5.40 (1H, -CH=C, cholesterol), 4.51 (1H, COO-CH-), 3.71 (2H, BocNH-CH_2), 3.11 (2H, Boc-NH-CH_2), 2.30 (2H, -CH_2COO-), 1.42 (9H，Boc), 2.23-0.61 (45H, cholesterol).
^{13}C NMR ($CDCl_3$, 75Hz): δ 172.7, 168.4, 159.1, 157.6, 139.7, 122.5, 108.8, 73.5, 56.6, 56.4, 55.9, 52.4, 49.7, 49.6, 42.1, 40.2, 39.8, 39.2, 38.4, 36.8, 36.6, 35.5, 35.5, 33.9, 31.7, 30.8, 28.8, 28.1, 27.5, 25.1, 24.5, 24.3, 24.1, 23.3, 23.2, 19.2, 18.4, 14.9.

ESI-MS：[M^+] = 657.6 (*m*/*z*), calculated: 657.5

2H-Cho-Arg:

^{1}H NMR ($CDCl_3$, 300Hz): δ 7.70-8.50 (Guanidyl-H, arginine), 7.0-7.60 (H, NH_3^+), 4.51 (1H, COO-CH-), 3.71 (2H, COO-CH-), 3.11 (2H, Boc-NH-CH_2), 2.30 (2H, -CH_2COO-), 1.42 (9H，Boc), 2.23-0.61 (45H, cholestanol).
^{13}C NMR ($CDCl_3$, 75Hz): δ 172.7, 168.4, 159.1, 157.2, 108.8, 72.9, 56.5, 56.4, 55.5, 53.8, 52.2, 49.7, 49.6, 44.4. 41.4, 40.1, 39.8, 39.2, 38.4, 36.8, 36.4, 35.3, 34.9, 34.1, 30.1, 28.8, 28.1, 27.6, 26.0, 24.5, 24.5, 23.3, 23.3, 20.9, 19.2, 18.4, 14.9.
ESI-MS: [M^+] = 659.6 (*m*/*z*), calculated: 659.5

Dios-Arg:

^{1}H NMR ($CDCl_3$, 300Hz): δ 7.70-8.50 (Guanidyl-H, arginine), 7.0-7.60 (H, NH_3^+), 5.40 (1H, -CH=C, diosgenin), 4.51 (1H, COO-CH-)，4.18 (2H，OCO-CH_2, diosgenin), 3.11 (2H, Boc-NH-CH_2), 2.30 (2H, -CH_2COO-), 1.42 (9H，Boc), 2.23-0.61 (45H, diosgenin).
^{13}C NMR ($CDCl_3$, 75Hz): δ 172.7, 168.4, 159.2, 157.3, 139.7, 119.1, 116.0, 108.7, 80.5, 73.9, 66.4, 65.8, 56.2, 52.2, 49.5, 41.5, 40.5, 40.1, 39.8, 39.2, 38.4, 36.8, 36.6, 34.8, 34.1, 32.0, 31.3, 31.1, 28.9, 28.7, 25.9, 25.0, 24.2, 19.6, 18.7, 17.5, 15.1.
ESI-MS: [M^+] = 684.5(*m*/*z*), calculated: 684.5

Tigo-Arg:

^{1}H NMR ($CDCl_3$, 300Hz): δ7.58-8.45 (Guanidyl-H, arginine), 6.88-7.58 (H, NH_3^+), 4.51 (1H, COO-CH-)，4.18 (2H，OCO-CH_2, tigogenin),3.11 (2H, Boc-NH-CH_2), 2.30 (2H, -CH_2COO-), 1.42 (9H，Boc), 2.23-0.61 (45H, tigogenin).
^{13}C NMR ($CDCl_3$, 75Hz): δ 172.7, 168.4, 159.2, 124.7, 108.7, 86.9, 73.9, 66.4, 65.8, 56.2, 56.2, 52.2, 49.5, 42.4, 41.5, 40.5, 40.1, 39.9, 39.2, 38.4, 36.8, 36.2, 34.8, 34.1, 31.8, 31.6, 29.1, 28.8, 27.7, 23.1, 22.7, 20.9, 19.2, 18.4, 12.5, 11.6.
ESI-MS: [M^+] = 686.5(*m*/*z*), calculated: 686.5

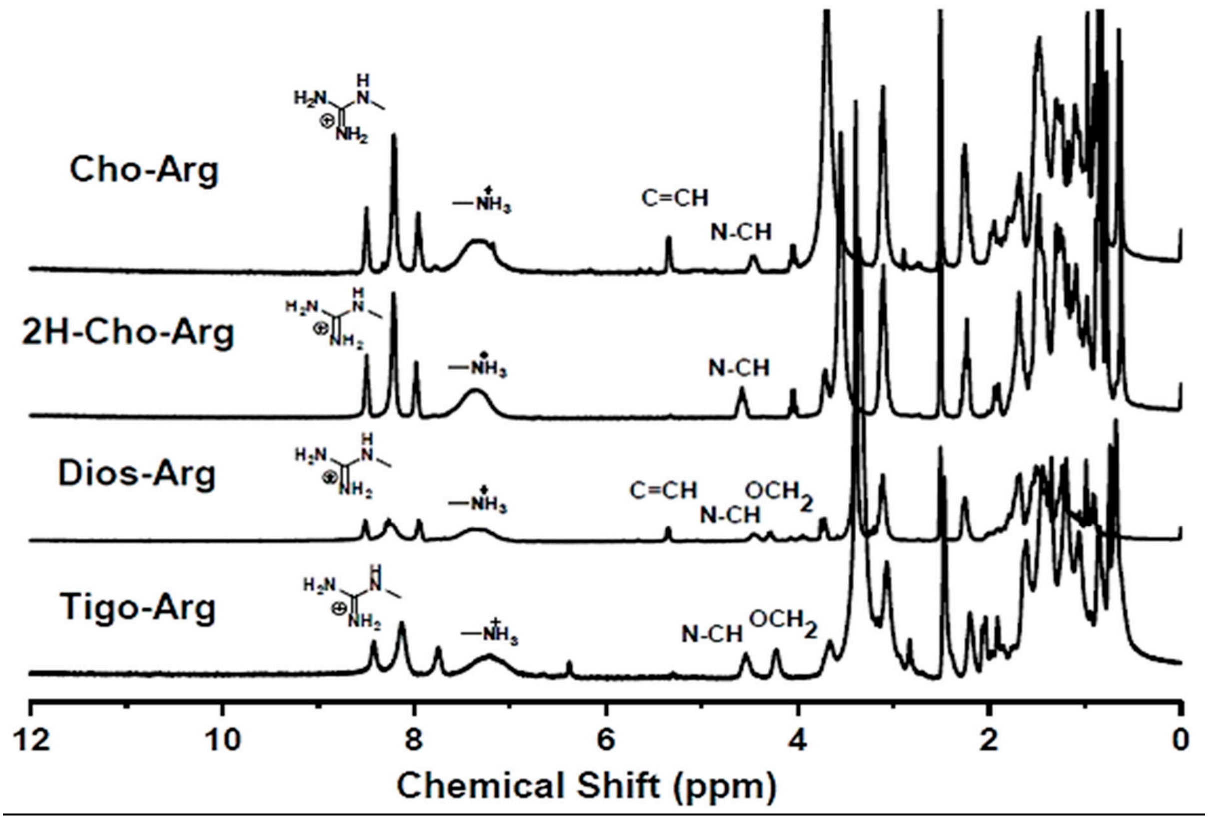

Figure S1b. ^{1}H NMR spectra of the steroid-based cationic lipids in d_6-DMSO solution.

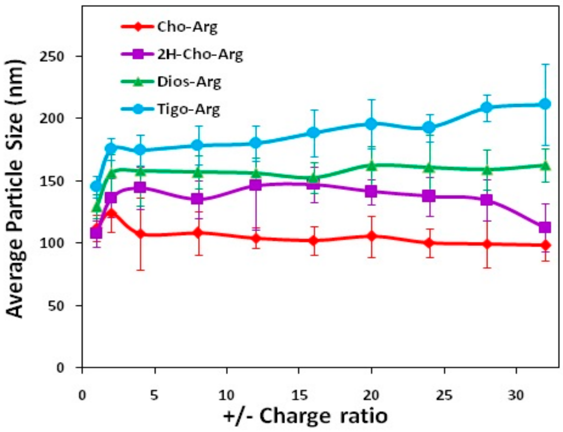

(a)

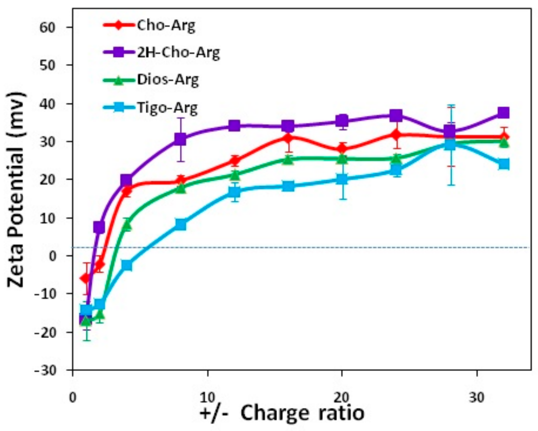

(b)

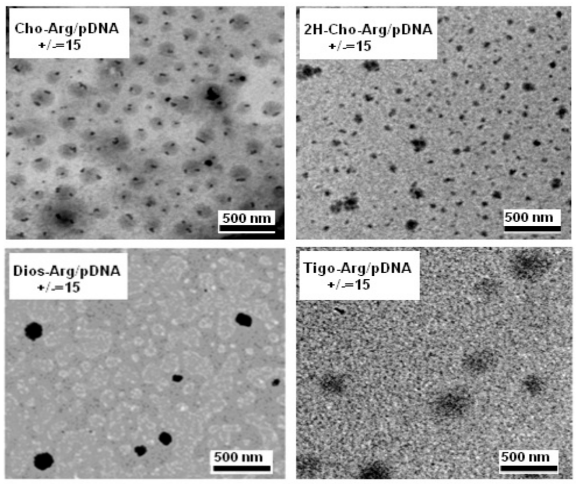

(c)

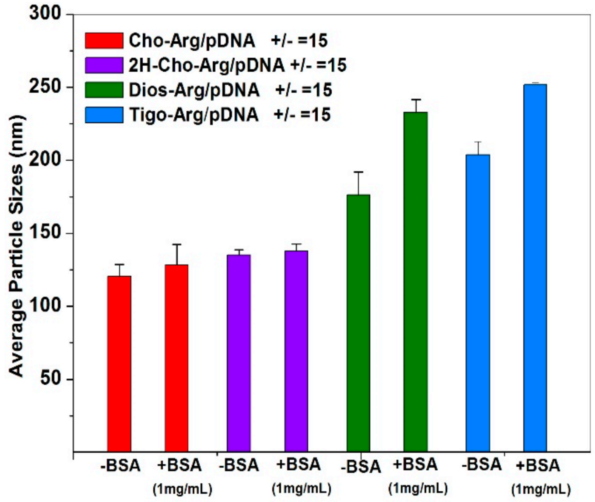

(d)

Figure S2. (**a**) Average particle size; and (**b**) zeta potential of the steroid-based cationic lipids/pDNA lipoplexes under various +/− ratios measured by DLS at 25 °C (λ = 633 nm, scattering angle: 90°). (**c**) TEM images of the lipoplexes (± = 15) at dried state. (**d**) The average particle size of the lipoplexes (± = 15) under the presence of BSA (1mg/mL).

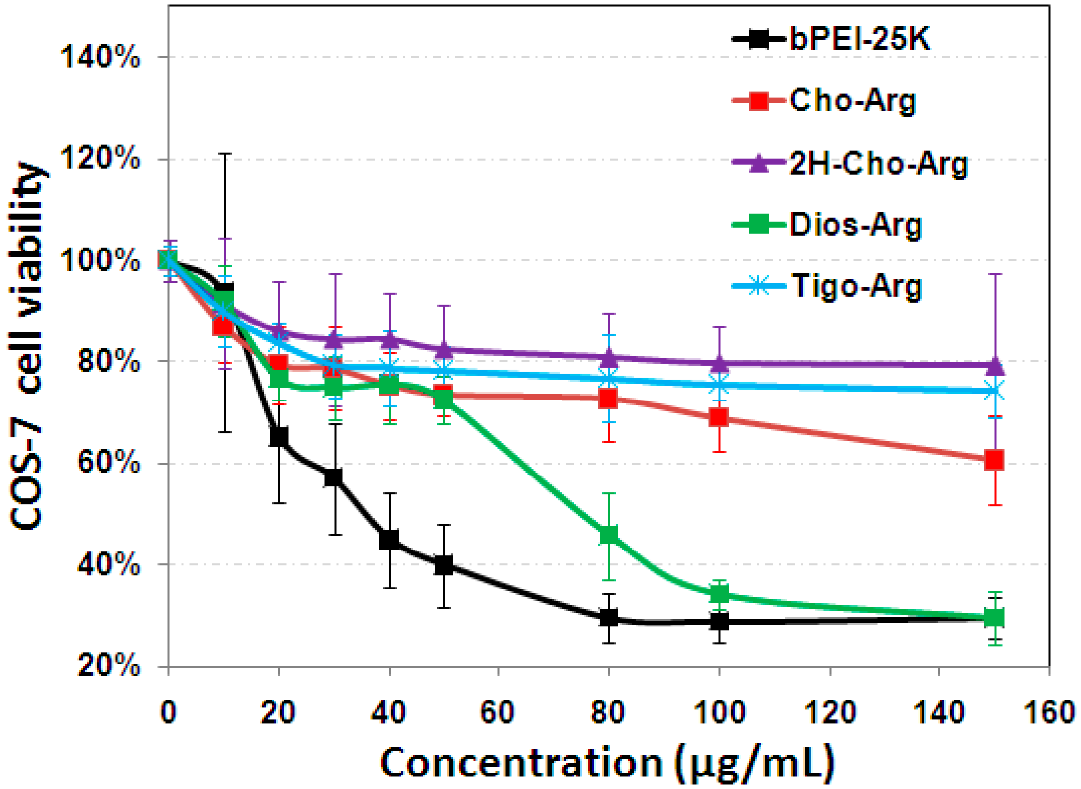

Figure S3. The cytotoxicity (MTT) assays of the steroid-based cationic lipids at various concentrations (from 0 to 200 µg/mL) in COS-7 cells.

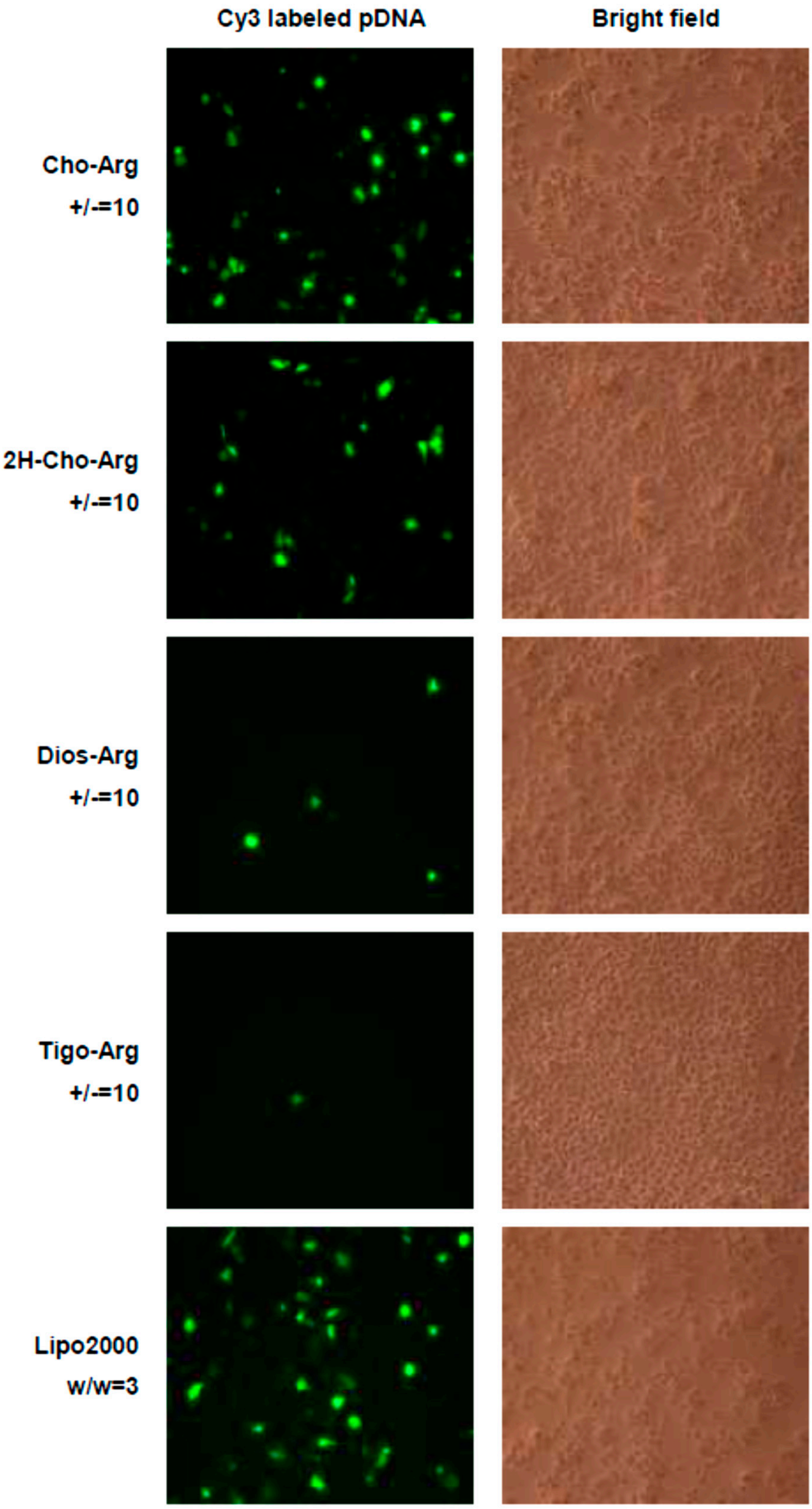

Figure S4. EGFP expression of the H1299 cell transfected with steroid-based cationic lipids/pEGFP DNA lipoplexes (± = 10) observed; commercially available Lipo2000 was used as the control.

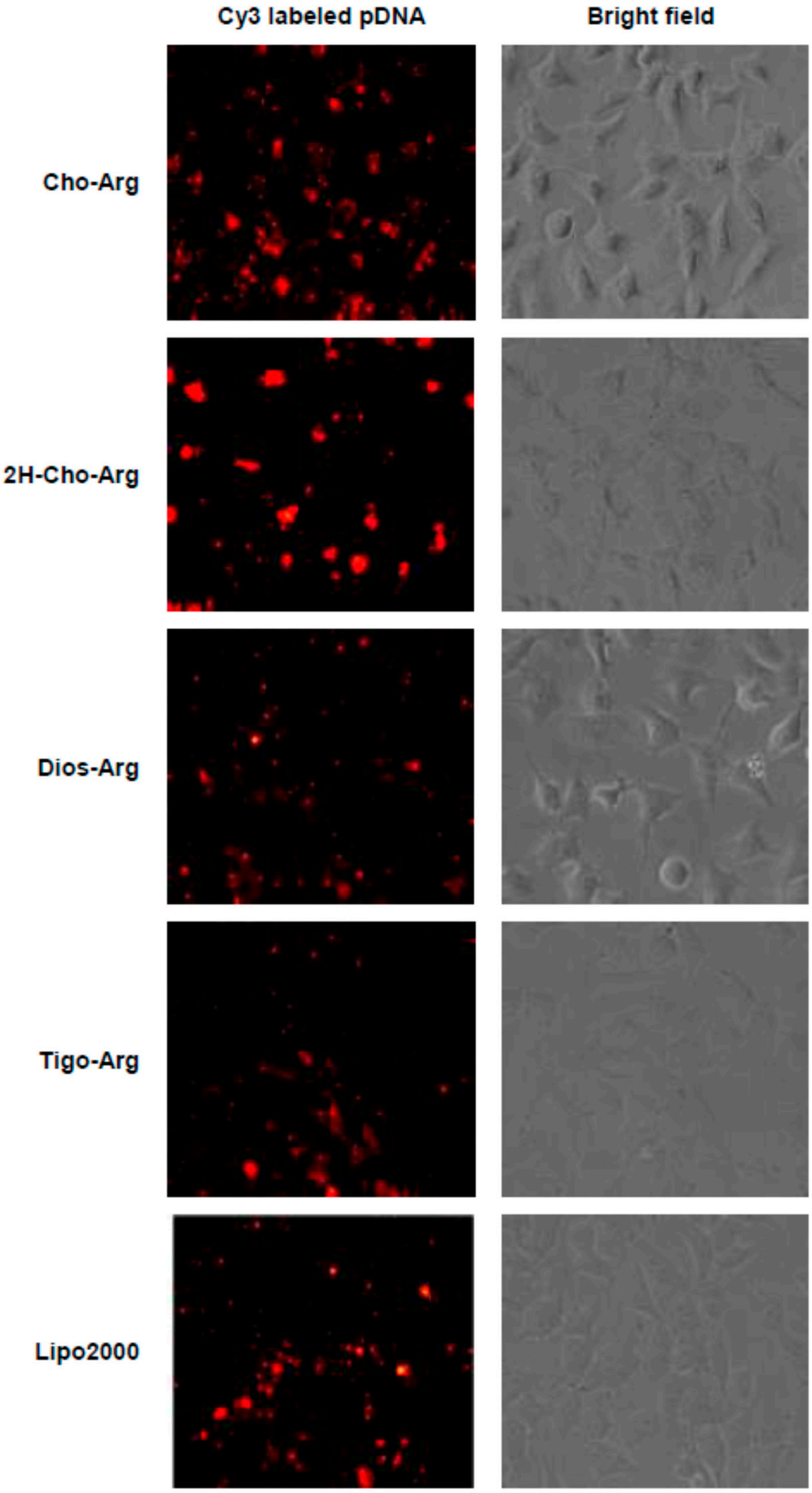

Figure S5a. Fluorescence images (400×) of the steroid-based cationic lipids/Cy3-pDNA lipoplexes after 4 h incubation in H1299 cells; Lipofectamine2000 (Lipo2000) was used as a control.

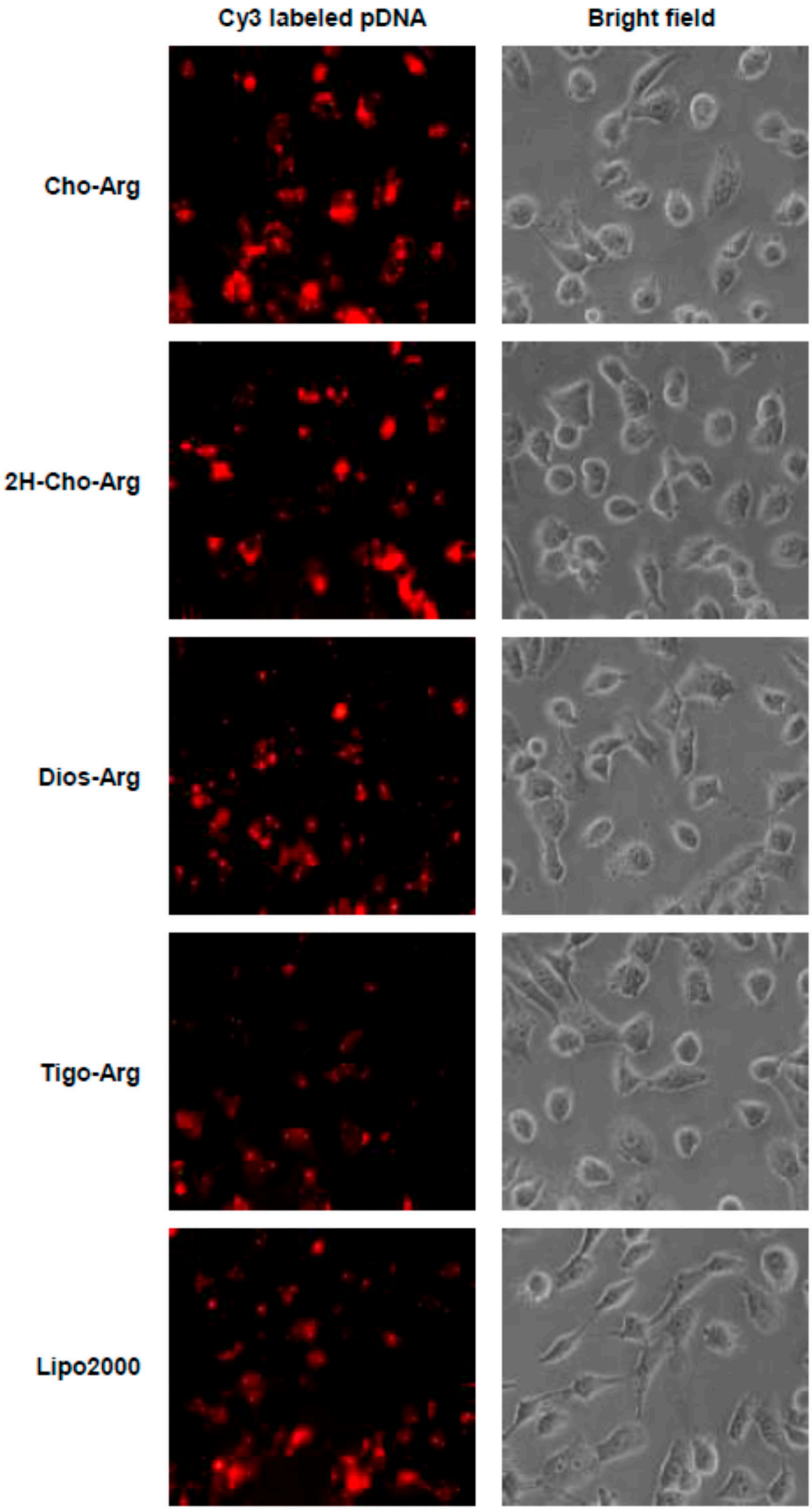

Figure S5b. Fluorescence images (400×) of cellular uptake of the Steroid-Arg cationic lipids/Cy3-pDNA lipoplexes incubated with the gene carriers for 24 h in H1299 cells; Lipo2000 was used as a control.

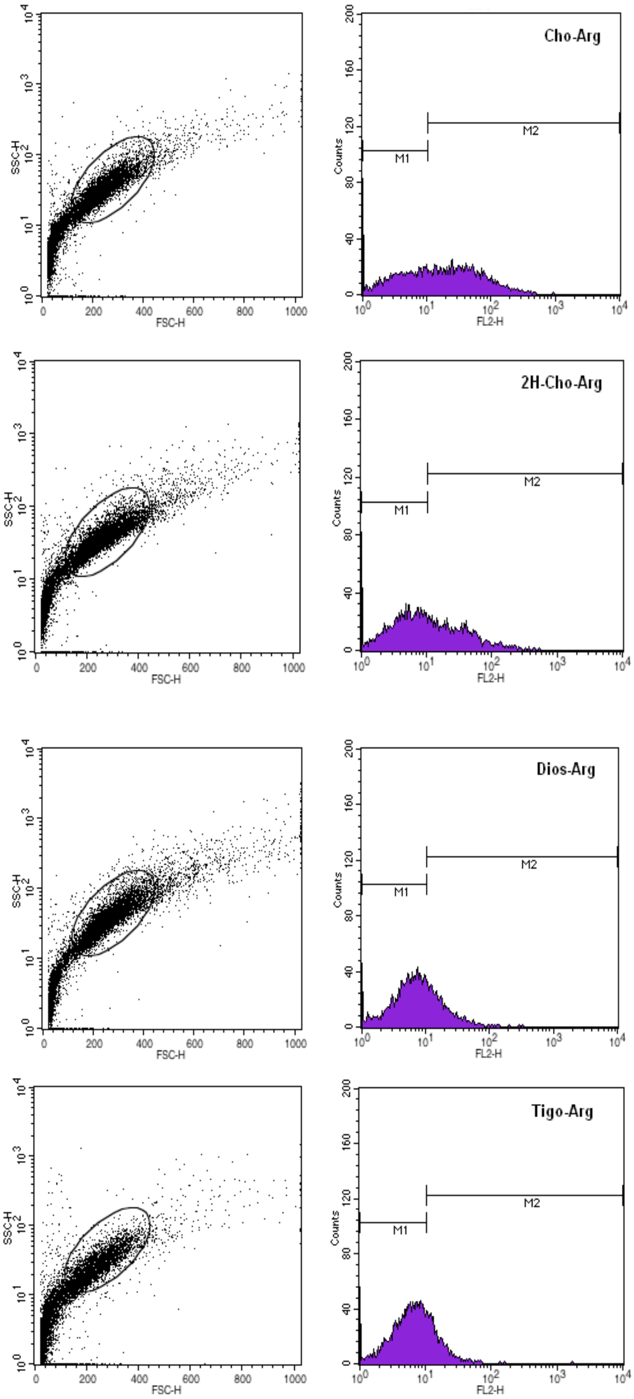
Cho-Arg
2H-Cho-Arg
Dios-Arg
Tigo-Arg
SSC-H
FSC-H
Counts
FL2-H
M1
M2

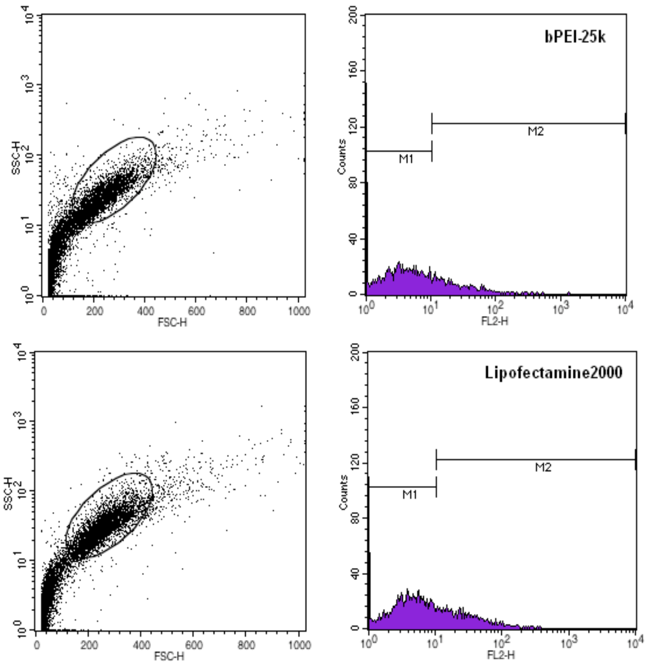

Figure S6. Original profiles of the intracellular uptake of the steroid-based cationic lipids/pDNA lipoplexes in HeLa cells analyzed by FACS; Lipo2000 and bPEI-25k were utilized as the controls for gene transfection in the optimized dose.
